# GDF15: A Hormone Conveying Somatic Distress to the Brain

**DOI:** 10.1210/endrev/bnaa007

**Published:** 2020-04-20

**Authors:** Samuel M Lockhart, Vladimir Saudek, Stephen O’Rahilly

**Affiliations:** MRC Metabolic Diseases Unit, Wellcome Trust-Medical Research Council Institute of Metabolic Science, University of Cambridge, Cambridge, UK

**Keywords:** GDF15, GFRAL, RET, obesity, cachexia, hyperemesis gravidarum

## Abstract

GDF15 has recently gained scientific and translational prominence with the discovery that its receptor is a GFRAL-RET heterodimer of which GFRAL is expressed solely in the hindbrain. Activation of this receptor results in reduced food intake and loss of body weight and is perceived and recalled by animals as aversive. This information encourages a revised interpretation of the large body of previous research on the protein. GDF15 can be secreted by a wide variety of cell types in response to a broad range of stressors. We propose that central sensing of GDF15 via GFRAL-RET activation results in behaviors that facilitate the reduction of exposure to a noxious stimulus. The human trophoblast appears to have hijacked this signal, producing large amounts of GDF15 from early pregnancy. We speculate that this encourages avoidance of potential teratogens in pregnancy. Circulating GDF15 levels are elevated in a range of human disease states, including various forms of cachexia, and GDF15-GFRAL antagonism is emerging as a therapeutic strategy for anorexia/cachexia syndromes. Metformin elevates circulating GDF15 chronically in humans and the weight loss caused by this drug appears to be dependent on the rise in GDF15. This supports the concept that chronic activation of the GDF15-GFRAL axis has efficacy as an antiobesity agent. In this review, we examine the science of GDF15 since its identification in 1997 with our interpretation of this body of work now being assisted by a clear understanding of its highly selective central site of action.

## Graphical Abstract

**Graphical Abstract:**
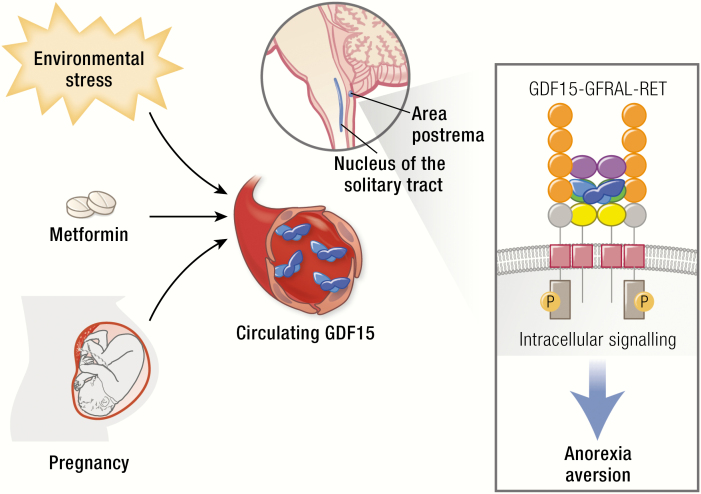

**Essential Points**

- GDF15 is a stress-regulated hormone that signals exclusively via the GFRAL-RET heterodimer.
- GFRAL expression is restricted to the area postrema and nucleus tractus solitarius.
- GDF15-GFRAL-RET signaling suppresses appetite in mice and nonhuman primates and is perceived as aversive by mice.
- GDF15 is implicated in a wide range of human disease states associated with weight loss and nausea, including cancer cachexia, chemotherapy-related nausea and vomiting, and hyperemesis gravidarum.​
- Metformin increases circulating GDF15 in humans and an intact GDF15-GFRAL-RET axis is required for the weight loss effects of metformin in mice.

Growth differentiation factor-15 (GDF15) is a member of the transforming growth factor- β (TGF-β) superfamily, which is secreted by cells exposed to a broad range of stressors. Its role in appetite and weight regulation was first postulated when it was observed that tumors overexpressing this hormone could induce cachexia in mice, which was ameliorated by an anti-GDF15 antibody. In spite of this, its primary physiological function and the molecular basis of its actions remained enigmatic until recently, when its receptor, the GDNF family receptor alpha-like proto-oncogene tyrosine-protein kinase receptor Ret (GFRAL-RET) heterodimer, was simultaneously identified by 4 groups working independently. GFRAL is expressed exclusively in the hindbrain and activation of the GFRAL-RET heterodimer suppresses food intake. These findings firmly establish the GDF15-GFRAL axis as a novel hormonal system regulating ingestive behavior and have invigorated interest in the translational relevance of this system. Here we review the cellular and molecular biology of GDF15-GFRAL and consider the emerging roles of this hormonal axis.

## Discovery of GDF15

GDF15 (then termed macrophage inhibitory cytokine-1 [MIC-1]) was discovered by the Breit group in 1997, who used subtraction cloning to identify genes that were relatively enriched in monocytoid cells treated with phorbol 12 myristate 13-acetate (PMA) to model activated macrophages (1). Several other groups independently cloned the same gene by a variety of different approaches, each coining their own term for this new peptide (2–6). In 2001, the Eling group identified what they called nonsteroidal anti-inflammatory drug (NSAID)-activated gene-1 (NAG-1) using subtractive cloning in NSAID-treated colon cancer cells and used sequence analysis to determine that MIC-1, placental transforming growth factor-β (PTGF-β), prostate-derived factor (PDF), placental bone morphogenic protein (PLAB), and NAG-1 all encoded the same gene (7). Common to all these reports was the attribution of this novel peptide, designated GDF15 in 2000, to the TGF-β superfamily based on a characteristic seven cysteine region. The purported function in these initial reports largely depended on the context of its discovery. For example, PLAB and PTGF-β were described as having potential important regulatory actions on embryogenesis in keeping with its placental origin, while MIC-1 was characterized by its ability to inhibit the secretion of tumor necrosis factor-α (TNF-α) from macrophages in response to lipopolysaccharide (LPS). The literature describing the biology of the GDF15-GFRAL axis, which we detail below, has grown exponentially over 20 years. The identification of the highly anatomically restricted expression of the receptor and the realization that many commercial preparations of GDF15 are contaminated with other bioactive peptides (8) have helped to bring clarity to the complex and confusing literature on GDF15 that has accumulated over 20 years.

## Molecular Biology of GDF15

### The GDF15 gene

**Figure 1.**
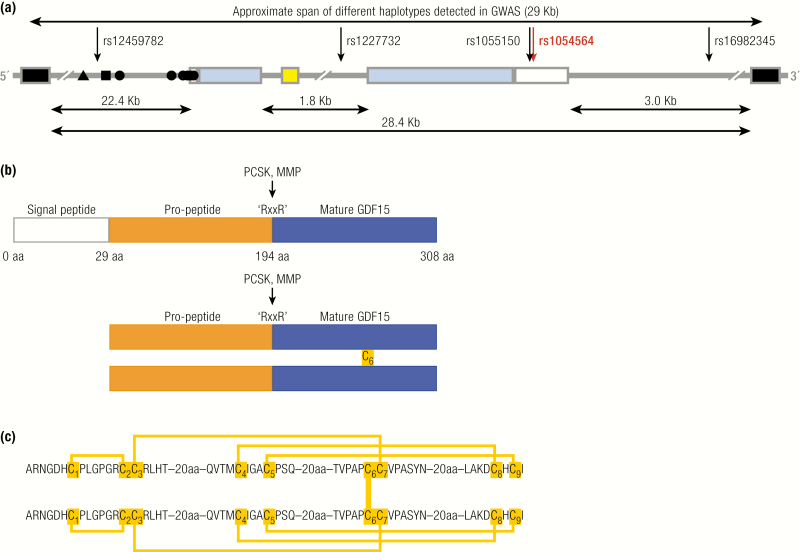
GDF15: Gene and protein structure. **A:** Schematic representation of the human genomic segment containing GDF15 between neighbor genes PGPEP1 and LRCC25. GDF15 in blue (coding exons) and white (UTR); the 3’ and 5’ coding exons of PGPEP1 and LRCC25, respectively, in black; miRNA miR-3189 in yello; and the experimentally established transcription factor binding sites in the promoter for ▲ CHOP, ■ P53, and ● Sp1/Egr1. Sense of the GDF15 transcript is from left to right. The arrows indicate the putative causal SNPs in 5 different haplotypes that might influence GDF15 transcription. The red arrow indicates the rs1054564 variant in the GDF15 3’-UTR that has been experimentally validated to alter GDF15 expression via altered miRNA binding (16). **B:** Schematic demonstrating the signal peptide (blue), propeptide (orange), and mature peptide of GDF15. Pairing of the 6th cysteine in 2 pro-GDF15 monomers forms a pro-GDF15 dimer, which can be secreted and bound to the extracellular matrix or proteolytic cleavage at an “RXXR” motif and can liberate the mature peptide, which is secreted and circulates as a bioactive homodimer. C: Schema illustrating the cysteine–cysteine pairing within, and between, GDF15 monomers.

The human GDF15 gene (Fig. 1A) is located on the forward strand of the short arm of chromosome 19 (19p13.11), flanked by the pyroglutamyl-peptidase I (PGPEP1) and leucine rich repeat containing 25 (LRRC25) genes upstream and downstream, respectively. This locus is contained within a region that is syntenic with a region on mouse chromosome 8 and is situated just over 1 Mb away from a primate-specific expansion that contains a large cluster of genes encoding zinc transcription factor proteins (ZNF) that arose early in primate evolution (9, 10). The mature transcript consists of 1200 bases and is coded for by 2 exons separated by a single intron (Fig. 1A). It has the notable feature, for an encoded peptide hormone, of containing 4 of the AU-rich instability motifs ‘ATTTA’ in its 3’-UTR, a feature more typical of classical cytokines (1). The GDF15 gene contains a TATA-like motif (TATAAA) upstream from the ATG start codon that is conserved between the human, rat and mouse genes (11). A number of transcription factor binding sites have been identified in the GDF15 promoter, including p53, early growth response-1 (EGR1), SP1-3, activator protein 1–2(AP1-2), Wilms tumor protein (WT-1), GATA binding protein-4 (GATA4), and nuclear factor kappa-light-chain-enhancer of activated B cells (NFκB) (11–13). The known positions of some transcription factor binding sites are indicated in Fig. 1A (14, 15). Further transcription factor binding sites likely exist. Humans inherit the GDF15 gene in at least 7 linkage disequilibrium-independent haplotypes (approximately 29 Kb) spanning the whole intronic and intergenic sequence between the neighboring genes and comprising many putative regulatory features (Fig. 1A).

In humans and old-world primates, a 73 base pair transcript encoding a microRNA (miRNA) miR-3189 is found in the GDF15 intron (Fig. 1A). Existing evidence suggests that this intronic miRNA is cotranscribed with the GDF15 gene (17, 18), but this has not been definitively proven. Indeed, many intronic mRNA have their own promoters located within their host gene (19, 20). Gain-of-function studies overexpressing either a proprietary miRNA mimic or a construct expressing the sequence corresponding to the pri-miRNA have suggested a role for miR-3189-3p as a tumor suppressor: expression of either construct suppressed the growth of a colon cancer and glioblastoma cell line in vitro and in tumor xenograft experiments (17, 18). It has also been suggested that miR-3189 autoinduces GDF15 (17, 21), but the mechanism underlying this effect is unknown. Detailed loss-of-function studies have not been published and further work is needed to determine the regulation and endogenous function of this miRNA.

### Structure, processing, and secretion of GDF15

GDF15 circulates as a 25kDa dimer linked by a single inter-chain disulphide bond. It is synthesized as a 308aa peptide consisting of a signal peptide, propeptide, and mature peptide (Fig. 1B). The membership of GDF15 in the TGF-β superfamily is conferred by high sequence homology and a conserved 9 cysteine region (1, 22). Eight of the 9 cysteines in the conserved domain form a cysteine knot, which functions to stabilize the mature GDF15 monomer. The orientation observed in the crystal structure of GDF15 is unique amongst all 9 cysteine TGF-β superfamily members in that cysteines 1 and 2 and cysteines 3 and 7 form disulphide bonds, whereas in the other 9 cysteine members cysteine 1 pairs with cysteine 3 and cysteine 2 pairs with cysteine 7 (Fig. 1C) (22). In the endoplasmic reticulum (ER), the 6^th^ cysteine in the 9 cysteine domain forms a disulphide bond with a free 6^th^ cysteine from another pro-GDF15 monomer to form a pro-GDF15 homodimer (Fig. 1B) (23). Between the propeptide and mature peptide, an RXXR motif exists at position 196. Following secretion from the ER proteolytic, cleavage occurs at this site to release mature homodimeric GDF15 from its propeptide (23). A yeast 2-hybrid screen and proteomic analysis have identified that matrix metalloproteinase-26 (MMP-26) (24) and paired basic amino acid-cleaving enzyme 4 (PACE4), respectively, can mediate this event (25). Hypothesis-driven analysis of the proprotein convertase subtilisin/kexin (PCSK) class of proteases (of which PACE4 is a member) demonstrated that PACE4, PCSK3, and PCSK5 could all facilitate the maturation of GDF15 via cleavage at its RXXR site in cardiomyocytes in vitro and in mice, in vivo (Fig. 1C) (26). Proteolytic cleavage at the C-terminus of the mature peptide by MMP14 has also been described (27).

Unlike other members of the TGF-β superfamily, GDF15 does not require an intact propeptide domain for processing and secretion, as when a GDF15 mutant lacking the propeptide is overexpressed the proteasomal inhibition has no effect on secretion of mature GDF15 and GDF15 does not accumulate intracellularly (23). This is in stark contrast to when the propeptide is overexpressed: in this case the propeptide accumulates when the proteasome is inhibited, suggesting that the propeptide domain facilitates recognition and disposal of incorrectly folded GDF15 (23). Interestingly, a GDF15–TGFβ1 chimera, where amino acids in positions 56–68 of GDF15 (comprising the major α-helix) were replaced with the corresponding amino acids in TGFβ1, could only be successfully secreted in the presence of the GDF15 propeptide (28). It has been inferred that this is evidence that the propeptide can facilitate correct folding and processing of GDF15 in certain circumstances, but the physiological relevance of this data is unclear.

In the processing of canonical TGF- β superfamily members, the prodomain remains noncovalently associated with the mature peptide and confers latency. In the case of GDF15 there is no evidence to suggest that it remains associated with its prodomain when processed and secreted. Instead, it is thought to circulate exclusively as an active homodimer. However, a number of transformed cell lines, including the choriocarcinoma cell line BeWo, the monocytoid cell line from which GDF15 was initially cloned, U937, and the prostate cancer cell lines, PC-3 and LNCaP, all secrete an unprocessed proGDF15 dimer where the propeptide domain has not been proteolytically cleaved (29). Interestingly, proGDF15 is rapidly secreted from cells and has a propensity to bind to the extracellular matrix, a property that mature GDF15 does not possess (29, 30). Xenografts formed in mice from a prostate cancer cell line expressing a proGDF15 mutant that cannot be processed exhibited marked upregulation of extracellular matrix (ECM)-bound GDF15. However, circulating GDF15 was not changed compared to mice with control xenografts (29). It is currently unclear if ECM-bound proGDF15 plays an important role in physiology, as the ability to secrete proGDF15 has been primarily ascribed to transformed cells. However, stroma-bound GDF15 can be detected in normal prostate and it is conceivable that ECM-bound proGDF15 could be a source of GDF15 that might be rapidly released by the action of proteases activated or released by cellular injury (29).

### Evolution of GDF15

**Figure 2.**
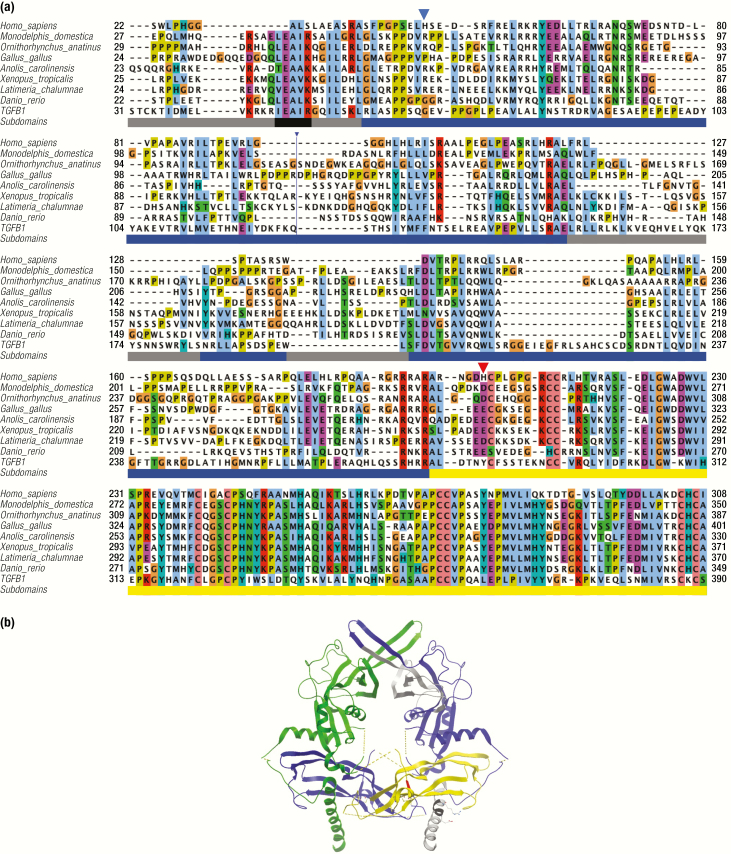
Evolution of GDF15 sequence and structure compared to TGF-β1. **A:** Amino acid alignment of GDF15 sequences from vertebrates and human TGF-β1. Selection and alignment are based on all available sequences and a single representative sequence is displayed for placentals, marsupials, monotherms, birds, reptiles, amphibians, coelacanths and fishes. Supplementary Figure 1 available at (31) displays more representative sequences in higher resolution. The bar under the alignment indicates the subdomains identified in the structure of TGF-β1: grey, regions absent in placental GDF15 (ie, the straitjacket helix at the N-terminus and the second part of the arm); blue, the rest of the propeptide; yellow, active ligand TGF-β1; black, conserved, helix-stabilizing motif in the strait jacket. The same color coding of the subdomains is used in the right-hand monomer in Fig. 3B. A large insertion 117–155 in the Gallus gallus sequence is not displayed (blue line). The residues affected by the common coding SNPs are indicated by triangles, H202 in red. **B:** Structure of human pro-TGFβ-1 (32). The subdomains in the monomer on the right is colored as described in Fig. 2A, the left is in green (propeptide) and magenta (ligand). The broken lines stand for the mobile loops invisible in the crystal structure. Grey color indicates the regions that are missing in the GDF15 of placentalia. The straitjacket is the N-terminal helix (bottom) where the black part indicates the strongly conserved, helix-stabilizing motif aEaaR (notably absent in placentalia—c.f. Fig. 2A) with the side chains of E and R forming a helix-stabilizing ion pair displayed. The position corresponding to the polymorphic H202D in human GDF15 is indicated in red.

As noted above, GDF15 exhibits important structural differences, which set it apart from its counterparts in the TGF-β superfamily. Examining the evolutionary history of this divergence is instructive. GDF15 likely evolved in the common ancestor of jawed vertebrates, as there is no clear orthologue observed in the genomes of the other 2 lineages of craniata, hagfish, lampleys, or lower vertebrates. Orthologues have been annotated in mammals, reptiles, amphibians, bony fish, and birds with a high level of conservation observed in the C-terminal region of the protein that represents the mature peptide. In contrast, the amino acid propeptide conservation is considerably lower, indicating significant remodeling and simplification during evolution (note progressive accumulation of deletions in vertebrate evolution) (Fig. 2A, Supplementary Fig. 1A available at (31)).

Unfortunately, the structure of pro-GDF15 is not yet known; however, the structure of the homologous TGF-β can be used to draw inferences (Fig. 2B). The amino acid alignment indicates that there are important differences in the amino acid sequence of GDF15 of placental mammals and its other orthologues (including nonplacental mammals). In the canonical members of the TGF-β superfamily there is a conserved N-terminal helix present in the propeptide domain, termed the “straitjacket” (Fig. 2C). It bonds with the “arm” domain of the propeptide and is responsible for noncovalent association of the propeptide with the mature peptide to form a latent complex (33). Rudiments of the straitjacket are present in all GDF15 proteins except those belonging to the placental mammals. The evolutionary simplification of the propeptide suggests that it is unlikely that GDF15 can form any stable association complex with its propeptide and explains why, unlike many TGF-β superfamily members, human GDF15 circulates as an active homodimer (1) and the so-called latent noncovalent complex known in other TGF family members has never been detected.

The significance of these changes in terms of evolutionary fitness is unclear at present.

The high expression of GDF15 in placenta and the high circulating levels observed in pregnant women suggest it is important in pregnancy (34). It may be argued that this is underscored by the changes observed in GDF15 in placentalia. However, it should be noted that genetic ablation of GDF15-GFRAL signaling in utero by knockout of GDF15 (35) or GFRAL in mice has resulted in no observable phenotype in pups or a mild, inconsistent reduction in the size of offspring (22, 36–38).

### Transcriptional regulation of GDF15

Circulating levels of GDF15 are raised in a variety of disease states, including cancer, atherosclerotic cardiovascular disease, and obesity. However, the specific molecular mechanisms underlying this upregulation are less well known and the data that do exist are largely from in vitro studies using transformed cell lines. One of the first transcription factors identified as a regulator of GDF15 was p53. Overexpression or pharmacological induction of p53 in lung, osteosarcoma, and breast cancer cell lines was shown to markedly upregulate GDF15, findings that have been confirmed in cancer cell lines from other tissues (39–41). This tumor suppressor protein is inactivated in 50% of cancers and is a key effector of the cellular response to genotoxic stress. At least 2 p53 binding sites are present in the GDF15 promoter (Fig. 1A), with 1 near the transcriptional start site (TSS) and a second that is over 800 bp upstream (42). Both binding sites have been shown to transactivate the GDF15 promoter but mutation of the 5’ binding site has a minimal effect on the ability of p53 to induce GDF15 expression, suggesting that only the proximate p53 binding site is of functional relevance (40, 42). The 3’ binding site may also be used to induce GDF15 expression by p63 (43). Importantly, treatment of cancer cell lines with the DNA intercalator doxorubicin robustly induces GDF15 in p53 wild-type cells but has no effect in p53-null cells, suggesting that p53-dependent upregulation is the primary mechanism via which GDF15 is produced in response to genotoxic stress (44). Other stimuli that have been suggested to utilize p53 to induce GDF15 include C-reactive protein in endothelial cells and vitamin D in prostate cancer cell lines (45, 46).

Another putative tumor suppressor protein that has been implicated in regulation of GDF15 is EGR1, a zinc-finger protein induced in response to growth factor signaling and DNA damage. A pharmacological agent that coordinately upregulated GDF15 and EGR1 in a colon cancer cell line also activated transcription at a region in the GDF15 promoter containing 2 experimentally validated EGR1 binding sites, suggesting that EGR1 is a direct transcriptional regulator of GDF15 (Fig. 1A) (13).

The integrated stress response (ISR) is an adaptive cellular response to a diverse array of cellular stressors. Cellular stress results in phosphorylation of the alpha subunit of eukaryotic translation initiation factor 2 (eIF2α). This remodels cellular translation, suppressing global protein synthesis while simultaneously activating transcription of an adaptive gene program via the ISR-effector, activating transcription factor 4 (ATF4). The net effect is the repartitioning of nutrients and the biosynthetic machinery to specific pathways that adapt cells to stress. Recently, our group have shown that this pathway is a potent regulator of GDF15 in a variety of cell types (47). The cellular stressors tunicamycin and thapsigargin robustly induced GDF15 mRNA, an effect that was not observed in mouse embryonic fibroblasts (MEF) with a mutated eIF2α isoform that is not phosphorylated in response to cellular stress, or in human cell lines treated with the pharmacological eIF2α inhibitor-integrated response inhibitor (ISRIB). In addition, GDF15 upregulation in response to tunicamycin and thapsigargin was reduced in MEFs with genetic ablation of ATF4 or human cell lines with siRNA-mediated knockdown of C/EBP homologous protein (CHOP), 2 key effectors of the ISR. This work is complemented by previous studies that have provided evidence for the ability of CHOP to bind to and activate the GDF15 promoter (Fig. 1A), suggesting that CHOP is the terminal effector which mediates GDF15 upregulation in response to the ISR (14, 48). An alternative mechanism of GDF15 upregulation by the ISR has been demonstrated by Baek and colleagues, who have shown that the ISR effector ATF3 is enriched at the GDF15 promoter in association with CCAAT-enhancer-binding protein-β (C/EBPβ) and is necessary for upregulation of GDF15 in response to capsaicin treatment (49).

The initial report describing GDF15 noted that it was induced by an array of proinflammatory stimuli (1). One candidate effector is the transcription factor complex NF-κB, which has been validated as a direct transcriptional regulator of GDF15 (50). There is an NF-κB binding site in exon 2 of GDF15 to which the NF-κB has been shown to bind using chromatin immunoprecipitation (ChIP) and an electromobility shift assay (EMSA) in mouse embryonic fibroblasts (MEFs) (50). Transcription from this promoter can be enhanced by treatment with TNF-α, and genetic manipulation of the NF-κB subunit p65 affects GDF15 expression (50).

The GDF15 promoter also contains a number of Kruppel-like factor (KLF) response elements. Recent work has demonstrated that the complement component C5a can upregulate GDF15 in a nonsmall cell lung cancer cell line via KLF5 (51). In the same report it was demonstrated that lysine acetyltransferase 2A (KAT2A) (AKA GCN5), a proposed regulator of peroxisome proliferator-activated receptor gamma coactivator 1-alpha (PGC-1α) activity in response to nutrient availability (52), forms a complex with KLF5 at the GDF15 promoter, acetylates KLF5, and enhances its activity at the GDF15 promoter. It is intriguing to speculate that KAT2A/GCN5 may modulate GDF15 expression in response to nutrient availability, but to our knowledge this hypothesis has not been formally tested.

The transcription factors implicated above are all potent inducers of GDF15 in response to cell stress. However, what regulates basal GDF15 expression is unclear. Do the above stressors occurring at physiological levels as part of tissue and organ homeostasis contribute to circulating GDF15 levels observed in health or is there constitutive expression of GDF15 in unstressed cells that can be suppressed? The transcription factors SP1 and SP3 have been suggested to be regulators of basal GDF15 transcription (53). GDF15 promoters with deletion of the 3 predicted Sp-family binding sites markedly reduced basal promoter activity, while ChIP and EMSA analysis confirmed binding of SP1 and SP3 to the GDF15 promoter (53). These experiments were all conduced in HCT116 cells, a colorectal cancer cell line; as such the relevance of these experiments to nontransformed cells is unclear.

A range of other transcriptional regulators of GDF15 have been proposed based on the correlation of transcription factor expression with GDF15 and the presence of putative transcription factor binding motifs in the GDF15 promoter but without definitive experimental evidence. For example, single cell RNA sequencing has been used to identify transcription factors that correlate with GDF15 expression in single cells from heart tissue, and a cell-specific gene regulatory network of GDF15 has been proposed based on this data (12). This work identified known regulators such as CHOP and ATF4 but also suggested novel candidates, one of which, GATA4, was validated in HL-1 cardiomyocytes: overexpression of GATA4 upregulated *GDF15* mRNA and ChIP demonstrated enrichment of GATA4 at the GDF15 promoter.

The discussion above has focused on positive regulation of GDF15; however, it has recently been demonstrated that the repressor of RNA polymerase III transcription MAF1 homolog (MAF1), a negative regulator of RNA III polymerase activity, binds to the GDF15 promoter where it suppresses basal *GDF15* expression (54). Traditionally, RNA III polymerase transcribes small RNAs, whereas mRNA is transcribed by RNA II polymerase. However, there is an RNA III polymerase promoter element in the GDF15 promoter, and knockdown of MAF1 enhances *GDF15* expression (54). The authors went on to show that MAF1 knockdown enhances binding of RNA III polymerase to the GDF15 promoter which co-operatively regulates RNA II polymerase-dependent transcription of *GDF15* mRNA, potentially via the induction of chromatin looping.

An additional emerging negative regulator of GDF15 transcription is the transcription elongation factor SPT5. Canonically, SPT5 facilitates the transcription of stress and inflammation-related genes. However, this does not seem to be the case for GDF15, as SPT5 inhibition using a small molecule inhibitor potently induced GDF15 mRNA in vitro (55). The mechanism underlying this effect has not been explored.

### Post-transcriptional regulation of GDF15

A key feature of the ISR, which potently regulates GDF15, is the coordinated upregulation of adaptive proteins while global protein synthesis is inhibited. One mechanism that facilitates this process is the formation of mRNA stress granules, which protect cytosolic mRNA from degradation. In an elegant study conducted in colon cancer cell lines, Park and colleagues provided experimental evidence to implicate this mechanism in regulation of GDF15 (56). The data showed that following induction of ER stress with thapsigargin treatment, protein kinase C-α (PKCα) is activated and translocates to the nucleus where it can phosphorylate the RNA binding protein ELAV-like protein 1 (ELAV1), which triggers ELAV1 nuclear exportation. ELAV1 then stabilizes *GDF15* mRNA by binding to AU-rich elements in its UTR. This process was partly dependent on CHOP-mediated suppression of peroxisome proliferator-activated receptor γ (PPARγ) expression. When CHOP was suppressed during ER stress using a ShRNA, PPARγ expression was enhanced and GDF15 induction was impaired. The authors found that PPARγ binds to PKCα and prevents its nuclear translocation, therefore preventing it from phosphorylating ELAV1 (56). In addition, ERK1/2 signaling was reported to prolong the association of *GDF15* mRNA with ELAV1 (56). The stimuli, mechanisms and kinetics of release of *GDF15* mRNA from these protective stress granules remains unclear and their importance in vivo and in untransformed cells has not been established. Delineation of these mechanisms will be important to allow modulation of this mechanism for the therapeutic regulation of GDF15.

RNA-binding region containing-1 (RNPC1) has also been suggested to regulate GDF15 via a post-transcriptional mechanism. Overexpression of RNPC1 in various cancer cell lines upregulated GDF15 mRNA and protein and prolonged the *GDF15* mRNA half-life (57). Mechanistically, RNPC1 is an RNA binding protein and was demonstrated to bind to AU-rich elements in the *GDF15* 3’-UTR via the RNPC1 RNA recognition motif; however it was not proven that this interaction was necessary for RNPC1 dependent upregulation of GDF15.

### Sites of expression of GDF15

Data from publicly available expression atlases suggest that GDF15 is expressed at relatively low levels in the basal state in most tissues. The placenta, prostate, and some of the abdominal viscera are relative exceptions to this with relatively high levels of protein and mRNA expression (58). Immunohistochemical analysis of these tissues in rodents suggest that GDF15 is predominantly seen in epithelial cells and macrophages but is not highly expressed in mesenchyme (3). In tissue injury, however, GDF15 can be markedly upregulated in response to cellular stress. For example, basal expression of GDF15 in muscle is low but can be markedly induced in response to energetic stress due to genetic deletion of the *Crif1* gene, which results in mitochondrial dysfunction (14). Similarly, in the adult rat brain, GDF15 is only expressed in the epithelium of the choroid plexus; however, following brain injury, GDF15 is induced in other regions and cell types, including neurons (59). It appears that GDF15 can be induced in most cell types in response to stress in vitro. In vivo, the situation may be somewhat more nuanced with specific cell types predisposed to producing GDF15 in response to local stress. For example, in response to phenformin treatment GDF15 is induced in perivenular hepatocytes in zone 3 of the liver acinus (60). Similarly, metformin treatment induces GDF15 in crypt enterocytes in the intestine (60). It may be an intrinsic feature of these cells that occurs in response to a range of stressors or it may reflect a cell type specific vulnerability to particular stimuli—in this case biguanides. GDF15 is also highly expressed in activated macrophages and upregulation of GDF15 expression at a tissue level could be explained by its content of infiltrating immune cells. This may be the case in adipose tissue of high fat diet (HFD)-fed mice (47) and in lung tissue of mice exposed to cigarette smoke (61).

A number of molecular mechanisms have been implicated in the regulation of GDF15 in response to stress, some of which we have discussed above. However, the molecular basis underlying high GDF15 expression in healthy placenta is unclear. High GDF15 expression seems to be an intrinsic property of the human trophoblast, as in vitro models of human trophoblasts, such as the choriocarcinoma cell line BeWo and placental organoids, exhibit high GDF15 expression independent of the milieu of human pregnancy (34, 62). Better understanding of the molecular mechanisms underlying the high constitutive, cell-autonomous expression of GDF15 in these cell types may aid future attempts to stimulate or repress endogenous GDF15 for therapeutic purposes.

### What physiological states or environmental agents are associated with changes in circulating GDF15 levels?

#### Technical and analytical considerations

A commercially available ELISA development set and an optimized “Quantakine” ELISA are available from R&D systems and have been widely used to measure circulating GDF15 concentrations in humans (63–73), and an immunoradiometric assay (also using reagents from R&D systems) has been validated and used in a number of clinical biomarker studies (74). We use an in-house assay developed from the Duoset kit available from R&D Systems (47, 60). A validation study of an electrochemiluminescence immunoassay from Roche, which has also been widely used, has been published (75). Using the immunoradiometric assay described above, GDF15 was shown to be stable in whole blood and serum at room temperature for at least 48 hours and over several freeze-thaw cycles (74), findings that are in agreement with unpublished validation data derived using our in house assay in human serum. Less data is available regarding available assays to measure circulating GDF15 in mice. However, an ELISA development kit from R&D Systems has been used by our group, and others, to measure circulating GDF15 in mice (47, 60, 76).

Changes in GDF15 expression in whole tissue at the mRNA and protein level are often reported. However, a number of reports have described changes in GDF15 at the tissue level but have not observed any alteration in circulating GDF15 (77, 78). Discordant changes in *GDF15* transcription and translation have been observed (79) and proGDF15 protein can be retained locally in the tissue matrix and not secreted into the bloodstream (29, 30). Therefore, it should be borne in mind that changes in local GDF15 expression may not result in biologically relevant changes in circulating GDF15.

#### Circulating GDF15 values in health

A broad normal range has been defined in healthy adult blood donors ranging from approximately 200 pg/ml to 1200 pg/ml (80–82) and exhibits a diurnal variation. It is markedly elevated at birth at concentrations of 3000 pg/ml, declining to levels within the healthy adult normal range within the first 4 months of life (67). The physiological significance of changes in circulating GDF15 from an individual’s baseline to a higher or lower level within the normal range is unclear.

### Nutritional states

#### Acute feeding.

In healthy human volunteers undergoing a standard glucose tolerance test where 50 g of glucose is consumed as a drink, GDF15 levels were not significantly altered for up 2 hours after ingestion (47). Similarly, in healthy volunteers, 5 mixed meals of various macronutrient content resulted in fluctuations in GDF15 between approximately 90% and 110% of baseline values, a degree of change that was not different than expected diurnal variation (83), and is in agreement with our own findings from mixed meal tolerance tests (47).

#### Undernutrition.

Short- to medium-term imposed caloric deficits have modest or no effect on GDF15 levels. A 24 hour fast in mice had no effect on circulating GDF15 levels despite 20% weight loss (47). In humans, restriction to 10% of estimated daily energy requirements for 2 days resulted in a modest increase of just over 25% of baseline levels. More prolonged calorie deprivation where healthy volunteers underwent total calorie restriction for 7 days led to a more pronounced increase in GDF15, with levels peaking at almost double baseline values after 48 hours before gradually returning to baseline (47). Restriction to 1000 Kcal/Day for 28 days, in volunteers suffering from obesity, resulted in a small, statistically significantly increase in GDF15 (47). Overall, the modest changes in circulating GDF15 in response to undernutrition are of uncertain biological significance and remain well within the accepted normal range.

#### Overnutrition.

In humans, sustained caloric excess consisting of high-fat feeding for 7 days or an additional 40% of weight maintenance energy requirements for 8 weeks did not alter circulating GDF15 (47). Similarly, 1 week of HFD-feeding in mice did not alter circulating GDF15.

In contrast, prolonged HFD-feeding of mice resulted in progressive elevation in plasma GDF15 from 4 weeks of HFD feeding which continued until 16 weeks of HFD in total, when GDF15 levels were approximately 3 times higher than control and the study was terminated (47). In keeping with these findings, GDF15 is clearly elevated in human and rodent obesity, which is a consequence of sustained overnutrition (47, 84, 85). Measurement of *Gdf15* mRNA expression by qPCR and microarray has demonstrated that *Gdf15* is increased in obese rodent liver and white and brown adipose tissue (47, 84).

Our group have shown that *GDF15* mRNA was found to correlate positively with a specific macrophage transcript, *Emgr1* (EGF-like module-containing mucin-like hormone receptor-like 1, encoding F4/80), suggesting that the elevated GDF15 may be elaborated from macrophages infiltrating adipose tissue (47). Adipose tissue inflammation is a feature of adipose tissue failure and, as such, increased GDF15 expression in obese rodent liver and adipose tissue may represent adipose tissue failure and subsequent hepatic injury by deposition of ectopic adipose tissue. Indeed, GDF15 is independently associated with HOMA-IR (85) and predicts the development of diabetes (86)—consequences of adipose tissue failure. Interestingly, GDF15 has been found to be markedly increased at the mRNA and protein level in mouse brown fat following 24 hours of cold exposure (77). While this did not translate to changes in circulating GDF15, it does suggest that alterations in adipose tissue phenotype could alter GDF15 expression in the absence of obesity associated adipose tissue dysfunction.

### Imbalanced amino acid diets

Diets deficient in essential amino acid content induce anorexia, a specific aversion to the deficient food-stuff and preference for foods containing the deficient amino acid in question (87). In keeping with the aversive properties of GDF15 and its regulation by cellular stress, mice fed a lysine-deficient diet exhibit marked increases in hepatic *Atf4*, *Ddit3* (encoding CHOP), and *Gdf15* mRNA and a 2-fold increase in circulating GDF15, which was observed as soon as 1 hour after exposure to the lysine deficient diet and persisted for at least 4 hours (47).

In summary, the existing body of evidence suggests that GDF15 is not potently regulated by fasting or feeding but that it may change in response to specific nutritional deficits such as amino-acid imbalanced diets. In addition, GDF15 elevation in chronic overnutrition and its associated metabolic disease likely does not reflect homeostatic elevation of an anorectic signal but, rather ,it is an index of organismal stress ensuing from metabolic dysfunction.

### Intense exercise

Studies suggest that circulating levels of GDF15 are elevated after intense exercise, but the source of GDF15 and the molecular mechanism of its increased release are unknown. In one study, healthy volunteers were fed a eucaloric diet for 3 days and subsequently underwent exercise testing on a bicycle ergometer. After 60 minutes of exercise at 67% of VO2_max_, GDF15 was increased by ~34% and by 67% after a further 120 minutes of rest (from a mean of 215 pg/ml at baseline to a mean of 350 pg/ml) (70). More dramatic increases are seen in ultramarathon competitors with 4-fold increases observed in circulating GDF15 after completion of the race, with levels reaching a mean of ~2300 pg/ml (88).

It is important to note that not all studies have found an acute effect of exercise on GDF15. In 1 study investigating the effects of exercise on heart transplant recipients, neither high intensity interval training, nor sustained moderate intensity exercise, altered GDF15 levels (89). The reason for this discrepancy is unclear but interpretation of these findings is complicated by the fact that basal samples were taken 1 week before exercise.

Repeated exercise training may also influence GDF15 levels. Volunteers with obesity underwent an exercise intervention consisting of 1 hour of aerobic exercise a day, 5 days a week, for 12 weeks (90). Serum GDF15 was significantly increased after the exercise intervention, but the absolute change was small (~60 pg/ml). The heterogeneity in the GDF15 response to exercise is of interest as the change in GDF15 was significantly associated with change in fat mass. Moreover, in a post hoc analysis where volunteers were designated as responders or nonresponders based on changes in GDF15 levels, only responders (in whom GDF15 increased after the exercise program) had a significant reduction in visceral fat, beneficial changes in cholesterol, and improvements in insulin sensitivity. Thus, elevated GDF15 in response to exercise training is associated with a greater metabolic benefit derived from exercise; however, it should be noted that this conclusion is based on findings from a post hoc analysis.

The elevation in GDF15 with intense physical activity is notable as a transient suppression in appetite can be observed following intense exercise in some settings (91, 92). While one may speculate that GDF15 may mediate any suppression of appetite by exercise, it should be noted that the authors did not measure indices of appetite or energy intake in this study.

The source of GDF15 in these studies has not been thoroughly examined, but it would seem plausible that exercising muscle secretes GDF15 perhaps in response to changes in myocyte metabolism. Indeed, the unfolded protein response (UPR) arm of the ISR is activated in skeletal muscle in response to exercise in mice and may upregulate GDF15 in this context (93). However, a recent meta-analysis of human muscle transcriptomics data after exercise suggests at best modest changes in muscle *GDF15* expression in relation to exercise (94).

### Hypoxia of altitude

Living at higher altitudes is inversely associated with the prevalence of obesity and exposure to moderate, high, or extreme altitudes is associated with a reduction in fat mass and fat-free mass (95–97). The ascent to higher altitude exposes individuals to hypobaric hypoxia and induces compensatory physiological responses. GDF15 has been implicated in this response and has been shown to increase by over 50% of baseline levels after 24 hours at high altitude (64). In a study with prolonged exposure to high altitude a similar magnitude of change was observed and GDF15 remained elevated while at high altitude but normalized to prestudy levels after subjects returned to sea level (98).

Cellular sensing of hypoxia occurs via accumulation and transactivation of the hypoxia-inducible factors hypoxia inducible factor-1 (HIF-1) and HIF-2. Hypoxia and CoCl2, which stabilizes HIF, can upregulate GDF15 expression in vitro (47). In addition, HIF-independent pathways may upregulate GDF15 following exposure to high altitude. For example, it has been shown that ER stress is increased in tissues from non-natives living at altitude (99).

### Environmental toxins

We have previously hypothesized that GDF15, a stress-regulated hormone, is secreted in response to noxious environmental stimuli (100). Indeed, a machine learning approach investigating the effects of toxin exposure (drugs and industrial toxins) on rats have demonstrated upregulation of *GDF15* expression, primarily in the kidney (101). Interestingly, *GDF15* expression in the kidney also correlated with weight loss and reduction in food intake, suggesting that GDF15 upregulation may play a role in toxin-induced weight loss. GDF15 is also upregulated in human duodenal mucosa from patients suffering from cholera infection, although plasma GDF15 levels were not assessed (102).

The most compelling line of evidence supporting this hypothesis is that which suggests smoking increases GDF15 secretion. Smoking may suppress appetite and be used by individuals to control weight whereas smoking cessation results in weight gain (103, 104). GDF15 covaries with smoking status in large epidemiological studies (105), is increased in the airway epithelial cells of individuals who smoke (106, 107), and is directly induced by cigarette smoke exposure in vitro and in rodent models in vivo (61, 106–108). In 1 study, WT mice exposed to cigarette smoke had reduced adipose tissue weight compared to air exposed controls. In contrast, adipose tissue weight was actually increased in *Gdf15*^*-/-*^ mice exposed to cigarette smoke compared to their air-exposed controls. However total body weight was not different between air and cigarette smoke controls in either genotype (61).

### Ageing

Chronological age is strongly associated with GDF15 in adults. In a cohort of over 600 individuals ages 21–113, GDF15 was significantly associated with age (rho = 0.805) (109). In a proteomic study of 240 healthy adults ages 22–93, GDF15 was the protein most significantly associated with age (110). Importantly, GDF15 has been shown to change prospectively with age: in a longitudinal analysis, circulating GDF15 levels changed by 11% on average after 5 years of follow-up (105). Moreover, GDF15 has been demonstrated to be a biomarker of age that is conserved in humans and mice (111).

Ageing is associated with the development of a frailty syndrome defined as “a state of vulnerability to poor resolution of homoeostasis after a stressor event, as a consequence of cumulative decline in many physiological systems” (112). Physical frailty is characterized by unintentional weight loss; self-reported exhaustion; reduced physical activity, grip strength. and walk speed; and is associated with sarcopenia. The biological mechanisms of frailty are an area of active interest and unsurprisingly GDF15 has been implicated. Frailty was found to be associated with GDF15, independent of age, in older adults who had recovered from acute coronary syndrome (113). Indeed, the frailty syndrome is associated with anorexia and sarcopenia and has a number of biological correlates with cancer cachexia—in which there is a clear pathogenic role for GDF15.

A cellular correlate of organismal ageing is senescence, a programmed cellular response to various stressors resulting in exit from the cell cycle and the acquisition of a secretory phenotype characterized by the secretion of a diverse array of typically proinflammatory mediators and growth factors—the so-called senescent-associated secretory phenotype (SASP). A range of studies have identified GDF15 as upregulated in senescent cells, but a recent proteomics study has identified GDF15 as a “core” SASP protein, upregulated in senescence in 2 different cell types by all of the stimuli tested (114). Thus, limited insults resulting in low level senescence likely result in trivial alterations in GDF15. However, we hypothesize that the accrual of senescent cells over time results in the progressive elevation of GDF15, which is sensed centrally, resulting in appetite suppression and contributes to the development of frailty.

### Childhood growth

Abnormal levels of circulating GDF15 compared to control and changes of GDF15 within a cohort have been associated with alterations in childhood growth. In one cross-sectional study of patients with congenital heart disease, children with congenital heart disease and a failure to thrive had significantly higher levels of GDF15 compared to their normal weight controls (65). In a longitudinal study of children who were small for gestational age (SGA), GDF15 levels were similarly elevated in SGA patients and appropriate for gestational age (AGA) controls; however, GDF15 levels at 4 months were significantly lower than in the SGA group and circulating GDF15 was inversely associated with catch-up growth. Moreover, GDF15 levels at 4 months were found to inversely correlate with changes in fat mass at 24 months. The authors suggested that these observations may represent an adaptive suppression in GDF15 that acts to enable catch-up growth, but the absolute differences seen are small and of uncertain significance (67).

## Disease States Associated With Elevated Circulating Levels of GDF15

Given that GDF15 is potently regulated by cellular stress, it is not surprising that its circulating levels are markedly elevated in a number of disease states. Even modest elevations in GDF15 above the defined upper limit of normal (1200 pg/ml) have been associated with increases in all-cause mortality (115). As such, GDF15 has been the focus of intense interest as a possible clinically useful biomarker of disease.

### Cancer

Circulating levels of GDF15 are raised in a range of human malignancies, including malignant glioma (71), pancreatic cancer (116, 117), colorectal cancer (72, 80, 118), and prostate cancer (29, 119–121). In addition, GDF15 levels correlate with tumor progression through the adenoma—carcinoma sequence in colorectal tumors and are elevated in metastatic cancer relative to local disease (80, 118). A large proportion of the elevated circulating GDF15 is undoubtedly attributable to the high levels of expression in tumors. However, risk factors for cancer, such as smoking and age, also elevate GDF15 and may also contribute to the raised levels observed in patients with malignancy.

A subset of patients with cancer develop an anorexia/cachexia syndrome characterized by involuntary weight loss (122). Cancer cachexia is associated with reduced quality of life, impaired function, and is postulated to directly contribute to the poor prognosis associated with advanced cancers (122, 123). The role of GDF15 in cancer cachexia has been known for over a decade. Its anorectic actions were first identified using GDF15 overexpressing human prostate cancer xenografts in nude BALB/c mice (124). The investigators found that mice harboring GDF15 overexpressing tumors progressively lost weight, muscle, and fat mass and exhibited reduced food intake. In addition, the plasma level of xenograft-derived human GDF15 predicted weight loss within the group, with GDF15 overexpressing tumors while treatment with a monoclonal antibody to GDF15 prevented weight loss. In the same report the authors demonstrated that serum GDF15 levels were positively associated with the amount of weight loss in a longitudinal study of patients with advanced prostate cancer (124). A contemporary study has confirmed the role of GDF15 in mouse models of cancer cachexia (125). Cytokine and hormonal profiling at the transcript and protein level demonstrated GDF15 to be one of the most upregulated factors measured in various mouse models of cancer cachexia. Treatment with GDF15-blocking antibody was capable of preventing cachexia in seven separate xenograft models of cachexia (125).

In esophageal and gastric cancer, prechemotherapy circulating GDF15 was found to be elevated in patients who had lost weight at the cessation of chemotherapy (126, 127). In a separate cross-sectional study of esophagogastric cancer, plasma GDF15 at diagnosis was associated with reduced dietary intake in a univariate analysis and was modestly elevated in patients with >10% self-reported weight loss (128). Similarly, in a cross-sectional study of patients with lung cancer, GDF15 was associated with historical self-reported weight loss (73). In a cohort of patients with primary tumors from various sites, GDF15 was elevated in those that had objectively determined weight loss in the preceding 6 months and was inversely associated with lean body mass, fat mass, and grip strength but did not correlate with an index of appetite (129). Prior to the discovery of the GDF15 receptor, Borner et al. made the notable observation that neurosurgical ablation of the area postrema (AP), but not subdiaphragmatic vagal deafferentation, reduces the anorexia, weight loss, and sarcopenia observed in a rat hepatoma allograft model of cachexia which we now know to be consistent with a key role for GDF15 acting via its receptor, GFRAL (130).

### Atherosclerotic cardiovascular disease

Clinical studies have demonstrated that GDF15 is elevated in patients with subclinical vascular dysfunction, atherosclerosis, and in those who subsequently develop the complications of atherosclerosis (131–137). Whether or not the upregulated GDF15 observed in these states is derived from cells in atherosclerotic lesions and dysfunctional vascular cells or from other sources remains unclear. Indeed, in patients with stable coronary artery disease a number of coincidental risk factors for cardiovascular disease have been shown to significantly affect plasma GDF15 concentration (138). These limitations notwithstanding, GDF15 expression is elevated in subendothelial macrophages in atherosclerotic lesions in humans and mice: GDF15 expression colocalized with Ox-LDL containing macrophages, apoptotic, and p53-expressing macrophages in human atherosclerotic lesions (139, 140). Thus, GDF15 is expressed by stressed macrophages in atherosclerotic lesions. Other vascular cells can express GDF15 in vitro when subjected to stress but the importance of this in atherosclerosis in vivo is uncertain (141).

GDF15 is upregulated in infarcted human myocardium relative to remote, noninjured myocardium and is elevated in the blood of individuals after myocardial infarction (142–144). In addition, GDF15 is upregulated in experimental models of myocardial infarction in mice and in response to oxidative stress in cultured cardiomyocytes (142, 145). The source of GDF15 in this setting has been debated. As has been noted by other commentators (146), serum GDF15 does not correlate with infarct size as assessed by cardiac MRI, suggesting that GDF15 secretion is not released solely from infarcted myocardium in this setting (147). A small study using high-frequency sampling of blood from patients after acute coronary syndrome has illustrated the temporal trend in GDF15 following myocardial infarction. In patients who did not suffer a recurrence of acute coronary syndrome (ACS) in the year after the index event, GDF15 levels peaked within 7 days of the index event, with a median value of 2436 pg/ml, which declined and then stabilized to average levels of just over 1500 pg/ml, which persisted for at least 30 days (148). The elevations in GDF15 levels are thus relatively modest and comparable to levels seen after extreme endurance exercise (88) and could easily be explained by extracardiac tissue dysfunction or perhaps even by activated macrophages in inflamed, unstable atherosclerotic lesions or other inflammatory cells infiltrating the injured myocardium.

### Heart failure

GDF15 is elevated in chronic heart failure, which correlates with severity and predicts its development (149–155). GDF15 is similarly elevated in stable heart failure with and without reduced ejection fraction (156). GDF15 is also increased in acute heart failure, where it correlates with clinical features of decompensation, declines during convalescence and is prognostically useful (157, 158). The magnitude of elevation in heart failure varies according to severity, comorbidities, and clinical setting but GDF15 levels in the normal range (<1200 pg/ml) are uncommon. In a contemporary clinical trial of pharmacotherapy in heart failure with reduced ejection fraction median, GDF15 levels were just over 1600 pg/ml, with an approximate 10% change in GDF15 levels observed per increase in NYHA class (155).

GDF15 expression is increased in the myocardium of animal models of heart failure (78, 159). In a mouse, postmyocardial infarction model of heart failure, *Gdf15* is upregulated almost 20-fold at the mRNA level in the myocardium versus noninjured hearts, but plasma levels are not significantly different (78). Similarly, in a pressure overload model of heart failure, myocardial *Gdf15* transcripts are elevated >5-fold after 4 weeks, but plasma levels were not different to control (78). Consistent with these findings, there is strong evidence to suggest that in human heart failure, the source of circulating GDF15 is extracardiac in nature. In patients with end stage heart failure, the majority of whom had dilated cardiomyopathy, GDF15 levels were markedly elevated relative to healthy controls; however, GDF15 expression could not be detected in myocardial biopsies from the same participants by RT-PCR or by immunohistochemistry (160). Interestingly, left ventricular assist device implantation (LVAD), which offloads the failing heart and improves end-organ perfusion, markedly reduced plasma GDF15 levels in patients with end-stage chronic heart failure, with 75% of individuals having GDF15 levels in the normal range after 6 months of LVAD (160). Reductions in GDF15 after LVAD implantation were paralleled by reductions in AST and creatinine, suggesting that some of the reduction may be secondary to the alleviation of cellular stress in the liver and kidneys. Indeed, in a randomized controlled clinical trial of sacubitril/valsartan, which prevents adverse cardiac remodeling, reduces heart failure morbidity and mortality, and reduces indices of cardiac stress, GDF15 was not different in placebo and experimental arms (155). These findings led the authors to propose that, in the setting of chronic heart failure with reduced ejection fraction, GDF15 is an integrated biomarker of multiple co-morbidities rather than a specific index of cardiac stress or dysfunction.

The role of GDF15 in the pathogenesis of heart failure is poorly understood. Investigations of its function have largely focused on paracrine effects and cell-autonomous actions, which are discordant with our current knowledge of how GDF15 acts. However, elevated GDF15 may contribute to the anorexia/cachexia syndrome observed in chronic heart failure—so-called cardiac cachexia—which is an important determinant of patient well-being and clinical outcome (161). GDF15 is inversely correlated with body mass index (BMI) in patients with chronic heart failure in keeping with a pathogenic role in cardiac cachexia (150).

### Acute and chronic kidney disease

GDF15 predicts incident chronic kidney disease (CKD) (162) and decline in renal function in established CKD (69, 163). In addition, GDF15 predicts the development of acute kidney injury post cardiac surgery (164, 165) and after treatment of acute myocardial infarction with percutaneous coronary intervention or coronary artery bypass grafting (166). Importantly, the association of GDF15 with the development of CKD and acute kidney injury in these studies persists after adjustment for important covariates, including age and smoking.

Renal expression of *Gdf15* mRNA is increased in a mouse ischemia reperfusion model of acute kidney injury (167). Acidosis commonly occurs in renal failure and other primary pathologies that may be associated with kidney injury. In one study, experimental acidosis in mice upregulated expression of *Gdf15* mRNA in the medullary collecting duct >40-fold (168). Similarly, in biopsies of kidney allografts, *GDF15* is increased after reperfusion of the transplanted kidney (169). In addition, in a cohort of 24 patients with CKD plasma, GDF15 was positively correlated with *GDF15* mRNA levels in renal biopsies (r = 0.54, *P* = 0.01) (69). Thus, in response to both acute and chronic renal injury, GDF15 expression is upregulated in the kidney and levels of circulating GDF15 increase. Interestingly, renal transplantation in patients established on hemodialysis reduces but does not normalize circulating GDF15 levels, suggesting that elevated GDF15 in end-stage renal disease is only partly driven by the uremic milieu (170).

Like heart failure and cancer cachexia, it is likely that GDF15 is a pathogenic factor in the anorexia/cachexia syndrome observed in advanced CKD. The association of GDF15 with the renal cachexia syndrome has been studied previously and GDF15 was found to be inversely associated with BMI in ESRD (124). The effect of GDF15 on renal cachexia has not been tested experimentally, but given what is known about the function of the GDF15-GFRAL axis, it is highly likely that GDF15-GFRAL signaling at least partly mediates the anorexia observed.

### Mitochondrial disease

Mitochondrial disorders encompass a heterogenous group of clinical entities characterized by greatly impaired mitochondrial function due to mutations in either the mitochondrial or nuclear genome that impair the function of mitochondrially expressed proteins. Patients commonly exhibit myopathy, neurological disorders, and developmental delay. Kalko and colleagues undertook transcriptomic analysis of muscle biopsies from patients with mitochondrial DNA depletion syndrome caused by loss of function in the thymidine kinase 2 (*TK2*) gene. GDF15 was upregulated 150-fold (171). The authors went on to demonstrate markedly elevated circulating GDF15 in children with mitochondrial myopathies (mean: 3562 pg/ml, large range with max value almost 90 000 pg/ml), but not in nonmitochondrial myopathies such as the muscular dystrophies. The utility of GDF15 in mitochondrial disease has been confirmed in several studies where GDF15 has been shown to correlate with clinical and histopathological markers of disease severity, with extent of mitochondrial heteroplasmy and with response to nucleoside therapy (68, 172, 173).

The general concept, that mitochondrial dysfunction upregulates GDF15, is in keeping with a range of murine studies that have demonstrated markedly elevated levels of GDF15 in skeletal and cardiac muscle when mitochondrial oxidative phosphorylation function is genetically perturbed (14, 174, 175). Mechanistically, impaired oxidative phosphorylation induces the mitochondrial unfolded protein response and a CHOP-dependent induction of GDF15 (14). In a mouse model of mitochondrial myopathy driven by accumulation of mitochondrial DNA mutations, *Gdf15* is upregulated early alongside *Fgf21* in what the authors termed the “first-phase” of the mitochondrial ISR, which seems to be independent of ATF3, ATF4, and ATF5, as these were either not induced in this model or were induced after GDF15. Notably, the role of CHOP was not specifically examined (176). In a separate study, rapamycin treatment completely inhibited *Gdf15* induction in a mouse model of mitochondrial myopathy, implicating mTORC1 signaling (174).

Therefore, GDF15 is potently induced by mitochondrial stress in skeletal muscle in rodent models and circulates at high levels in human mitochondrial disease. It has been proposed that this represents a signal that is elaborated to promote stress resistance at the organismal level (14). While this remains to be firmly established, it is supported by evidence demonstrating altered metabolism in mouse models of mitochondrial myopathy that is dependent on GDF15 (14).

### Thalassemia

Thalassemias are genetic disorders caused by mutations in the genes encoding the peptide constituents of hemoglobin. Dysregulation of normal hemoglobin formation results in toxic aggregates of globin chains, excessive cellular stress, and subsequent red blood cell hemolysis. Compensatory erythropoiesis is ineffective and contributes to subsequent splenomegaly and iron overload.

The first study to examine GDF15 in the thalassemias was prompted to do so by findings demonstrating markedly increased GDF15 expression in erythroblasts as an in vitro model of erythropoiesis (63). The authors went on to demonstrate that GDF15 was markedly upregulated in both α and β-thalassemia—with mean values in β-thalassemia of 66 000 pg/ml, which is among the highest levels seen in any human disease. A marked elevation of circulating GDF15 in thalassemia have been confirmed several times (177–180).

This seminal study has shaped the perception of the function of GDF15 in iron homeostasis. An impressive negative correlation of serum GDF15 with a negative regulator of iron bioavailability, hepcidin, coupled with in vitro studies showing GDF15-dependent suppression of hepcidin expression by thalasemia serum led to the conclusion that a key endogenous function of GDF15 is to regulate hepcidin expression and thus iron bioavailability (63). Subsequent studies have shown a positive association between GDF15 and hepcidin in anemic patients. Moreover, GFRAL is not expressed in hepatocytes, the site of hepcidin production (22, 36–38). As such, it is unclear if GDF15 regulates Hepcidin expression and if it does, it is unlikely to occur via a direct action on hepatocytes.

### Acute infection

Moderate-sized cohort studies have demonstrated that GDF15 is elevated in critically ill patients with the acute respiratory distress syndrome (181), a large number of whom have an infectious illness, and in critically ill patients with sepsis (182–184). It has also been demonstrated that GDF15 may have prognostic utility in patients with sepsis, with elevated levels predicting mortality (182).

Elevated GDF15 in these patients could be driven by any number or combination of pathways that regulate GDF15; however, there does seem to be a specific induction of GDF15 in response to bacterial and viral infection in mouse models that is driven by *Gdf15* upregulation in the liver, kidney, and peripheral blood (183, 185). At a molecular level this could be driven by direct toll linked receptor-agonism by the pathogen, as has been suggested by an ex vivo mechanistic study using bone marrow derived macrophages (183). It is also conceivable that proinflammatory cytokines such as interleukin-1β (IL-1β) and TNF-α act via NF-κB on solid organ parenchyma or infiltrating immune cells (50), though the role of this pathway in sepsis has not been tested. Energetic stress driven by hypoperfusion and altered availability of metabolic substrate could also play a role, but again this has not been explored.

## Purported Actions of GDF15 Prior to the Discovery of GFRAL

Up to this point we have deliberately limited our discussion of the function of GDF15. A multitude of actions have been attributed to GDF15 since its initial discovery in the late 1990s (comprehensively reviewed in (82, 186)). The majority of these actions are derived from in vitro studies, often attributed to GDF15 activity at 1 or other members of the family of TGF-β receptors and were consistent with GDF15 acting as an autocrine or paracrine factor at its site of production. The discovery that the sole receptor for GDF15 is GFRAL-RET and that GFRAL has a very restricted tissue distribution calls into question the interpretation of many previously published observations. Importantly, it has recently been reported that commercially available recombinant GDF15 made in eukaryotic cells is not infrequently contaminated with TGF-β (8). The latter is known to be able to exert potent biological actions at concentrations as low as the femtomolar range. This may explain many of the published findings that have involved the study of the effect of recombinant GDF15 on cells. That said, it is important to carefully examine the prior literature that involved manipulation of endogenous levels of GDF15 and/or its blockade by antibodies, particularly if they suggest possible actions of GDF15 that might not be mediated through a receptor expressed only in the hindbrain

### Anorexia and weight regulation

Pioneering work by Breit’s group established that GDF15 was a potent anorectic factor in the context of cancer and identified its ability to prevent diet-induced obesity in mice long before the discovery of GFRAL. Using transgenic mice that overexpress GDF15 in macrophages (*Gdf15*^*Csf1R*^), Breit and colleagues demonstrated that *Gdf15*^*Csf1R*^ mice ate less food and had reduced weight compared to their wild-type controls (187). They went on to show that high-fat diet fed *Gdf15*^*Csf1R*^ mice have reduced fat mass and improved glucose tolerance. These findings were consistent with previous studies from the Eling lab in chow-fed transgenic mice overexpressing GDF15 (188) and with Breit’s own work demonstrating that *Gdf15*^-/-^ mice are heavier than syngenic wild-type controls (189).

Subsequent studies from the Breit group identified the anatomical site of action of GDF15. They demonstrated that GDF15 activated neurons in the AP, the medial division of the nucleus tractus solitarus (NTS), and the dorsal motor nucleus of vagus (DMX) relatively rapidly (60 minutes), consistent with a direct effect on these neurons. Ablation of the NTS and AP using microaspiration completely prevented the anorectic actions of GDF15 treatment (190).

It should be noted that work from the Eling group suggests that mice with transgenic overexpression of GDF15 may have a reduced weight independent of food intake. Similar to the Breit group, Eling et al found that mice that overexpressed human GDF15 were protected from diet-induced obesity and dysglycemia (191). Adipose tissue inflammation and average adipocyte size was also reduced in transgenic mice. The authors felt these findings could not be explained by differences in food intake alone, as food intake over a 10-day period was not different between wild-type and animals overexpressing GDF15. Rather, they reported that the transgenic mice were more metabolically active, with enhanced heat production and oxygen consumption using indirect calorimetry. Brown adipose tissue weight was actually lower in the transgenic mice but thermogenic gene expression was increased in both white and brown adipose tissue, suggesting that the changes in energy expenditure were driven by a global shift in adipose tissue metabolism rather than expansion of brown adipose tissue depots.

It is worth commenting that food intake in this study was normalized to body weight, and the validity of this approach has been challenged (192). Moreover, pair feeding studies have demonstrated that the effects of short-term treatment with exogenous GDF15 on body weight are dependent on food intake (37, 38). These considerations notwithstanding, the effects of elevated GDF15 throughout the life course may not be the same as short term treatment.

### Cancer biology

In chemical models of colon and lung carcinogenesis, transgenic overexpression of human GDF15 suppressed tumor formation (188, 193). Similarly, in the *Apc*^*min/+*^ model of intestinal neoplasia, where mice develop spontaneous intestinal adenomas, mice harboring a copy of the GDF15 transgene exhibited a reduction in the number of adenomas (188). In keeping with an antitumorigenic action of GDF15, GDF15 knockout increases mortality, tumor number, and tumor size in a mouse model of spontaneous prostate adenocarcinoma (194). In the same model of mouse prostate cancer overexpressing GDF15 in myeloid cells (*Gdf15*^*Csf1R*^ mice described above) that exhibit a reported elevation of circulating GDF15 between 10 and 90-fold, reduced local tumor burden as expected, although the rate of metastasis was unexpectedly increased (195). Thus, except for a dissenting report that found no change in tumor incidence, size, or invasiveness in GDF15-KO mice (196), experiments with genetic manipulation of GDF15, which alter GDF15 expression before the manifestation of cancerous pathology seem to support an antineoplastic function of GDF15.

A number of tumor xenograft studies have been used to examine the role of GDF15 in cancer; however, the results have been less consistent. These studies use genetic manipulation of GDF15 in the grafted tumor cells and, therefore, GDF15 expression remains unchanged in the host animal until graft injection. Overexpression of GDF15 in xenografts has been shown to reduce graft size and incidence by some investigators (7, 197, 198), whereas other investigators reported similar findings using RNAi-mediated knockdown of GDF15 (50, 71, 199). The discrepancies may be related to the origin of the primary tumor, genetic heterogeneity in cell lines used, and other technical considerations, such as number of tumor cells implanted. Regardless, it is clear that manipulating GDF15-GFRAL-RET signaling at the same time as tumor implantation (via tumor GDF15 knockdown or overexpression) is different to whole body knockout or overexpression of GDF15 from a transgene throughout the animal lifespan.

### Regulation of lifespan

In a study of lifespan in female mice overexpressing human *GDF15*, the Eling group determined that elevated circulating GDF15 prolonged life in both chow and HFD-fed animals studied for over 95 weeks (200). The transgenic mice lived for >15 weeks longer than their wild-type counterparts and exhibited elevated growth hormone (GH) levels and reduced insulin-like growth factor-1 (IGF-1) levels. This pattern led the authors to hypothesize that GH resistance could explain the changes in lifespan. However, they could not find change in any additional evidence to support this hypothesis; as GH receptor expression was unchanged in transgenic mice, GDF15 treatment did not alter GH signaling in vitro and downstream effectors of GH signaling were not different in wild-type and transgenic mice (although measured in the basal state). Contrary to other studies, food intake was not significantly different between the GDF15 overexpressing and wild-type animals, but it should be noted that food intake was normalized to bodyweight. Bioinformatic analysis of microarray data suggested that mTOR-dependent signaling was reduced in white adipose tissue from the transgenic animals. Consistent with this phosphorylated IGF-1R, AKT serine/threonine kinase (Akt) and mammalian target of rapamycin (mTOR) were all reduced in the basal state in white adipose tissue of transgenic mice overexpressing *GDF15*. Further studies of the effects of GDF15 on lifespan are needed to confirm these findings and examine the underlying mechanism.

### Renoprotective actions

GDF15 deficiency was reported to exacerbate renal injury in 2 separate mouse models of diabetic nephropathy (201). In a streptozotocin treated (STZ) model of type 1 diabetic nephropathy, *Gdf15*^*-/-*^ mice exhibited greater interstitial fibrosis and histological evidence of renal tubular injury than controls animals. In keeping with these findings, renal expression of proinflammatory cytokines was enhanced in *Gdf15*^*-/-*^animals. Similar findings were obtained when *db/db* mice lacking *GDF15* were studied.

In summary, prior to the discovery of GFRAL, a number of actions were attributed to GDF15, some of which we have reviewed here. All of these studies were hampered by the lack of knowledge of the GDF15 receptor and by the technical inability to measure circulating levels of endogenous mouse GDF15. In vitro studies used to support peripheral actions of GDF15 may be confounded by potential contamination of recombinant peptide with TGF-β and many are inconsistent with a GFRAL-dependent mechanism of action. The literature outlined above requires careful reappraisal in light of the discovery of GFRAL as the GDF15 receptor.

## GFRAL-RET Heterodimer the Receptor for GDF15

The discovery of the orphan receptor GFRAL as the cognate receptor for GDF15 by 4 separate pharmaceutical companies has shone a light on this once enigmatic cytokine and focused the attention of the scientific community on a novel anorectic hormone with therapeutic potential in disorders of energy balance.

The evidence that GDF15 signals exclusively via GFRAL and that the latter is solely expressed in the hindbrain currently appears compelling. It is, of course, possible that GFRAL might become expressed in some developmental stages of 1 or more species or some disease state that has not yet been studied, but at present it would seem appropriate to view all of GDF15’s biology through the prism of its primary action via a highly localized hindbrain receptor

Hsu et al screened approximately 4000 membrane-expressed proteins using a cDNA library, transfecting each construct individually into cells and treating them with FC-labelled GDF15 and a fluorescently-tagged FC-binding fragment (22). The authors reported that only GFRAL generated a robust fluorescent signal. On review of the source data, 50 constructs generated signals nominally larger than the pCDNA3 control construct. Only 10 constructs demonstrated a signal >2-fold of that of the control construct. The signal from the GFRAL-transfected cells was more than 1 order of magnitude greater than the signal from the well with the next highest signal and over 50-fold greater than the control construct. A similar approach undertaken by a separate group identified GFRAL and 4 other hits but only GFRAL was validated as a GDF15-binding partner in a second flow-cytometry-based assay (37). None of these 4 other hits generated signals above that of the pCDNA3 control in the Hsu et al data set. Using a separate, flow-cytometry-based approach screening all 2762 of the known single pass transmembrane receptors, Yang et al reported only GFRAL as a hit. Thus, 3 independently conducted screens by different groups identified only GFRAL as a GDF15 binding partner.

Separate to the screening approaches used to identify GDF15-GFRAL binding the homology between GDF15 and the GDNF family prompted a hypothesis-driven investigation of the binding of GDF15 to the GFRA-receptor family, including GFRAL and GAS1 (36–38). No interaction between GDF15 and any of the GFRA-receptor family, except GFRAL, was identified.

Approaches using transfection of single receptors would be liable to miss signaling via heterodimer receptors, such as occurs in the TGFβR family. Importantly, hypothesis-driven testing of the interaction between GDF15 and various combinations of the Type 1 and Type 2 TGFβR family using a radioligand binding assay and a commercial chemiluminescence-based assay found no evidence of interaction between GDF15 and any of the tested combinations of receptors (22, 37).

As the GDNF family of ligands have been reported to exhibit promiscuity with respect to receptor signaling, it was confirmed that no members of the GDNF family were capable of binding to GFRAL using competitive binding assays of biotin-labeled GDF15 (38). Bone morphogenic protein-9 (BMP9) and TGFβ1 were also tested and could not displace biotin-labeled GDF15 from GFRAL.

Thus, 4 separate groups working independently with related but distinct methodology have provided an extensive catalogue of evidence from cell-based assays to support the assertion that GDF15 and GFRAL bind exclusively to each other in vitro and have deliberately tested and refuted previously hypothesized GDF15 receptors. A caveat to consider is that this data does not exclude GDF15 bioactivity via nonmembrane-bound receptor mechanisms, indeed an intracrine role for full length, unprocessed GDF15, as a transcriptional modulator has been proposed and could explain some of the TGFβ-like activity of GDF15, which has been previously described (202, 203). Regardless of the cell-autonomous effects of intracellular, unprocessed GDF15, the molecular insight provided by these studies has illuminated GDF15 as a hormone that when secreted signals exclusively via the hindbrain-restricted receptor GFRAL.

### GDNF family receptor alpha-like (GFRAL) structure

GFRAL derives its name from GDNF-receptor alpha family, of which there are 4 bona fide members (GFRA1-4) and 2 slightly more distant homologs—GFRAL and growth arrest specific-1 (GAS1). The GFRAs are glycosylphosphatidylinositol (GPI)-linked proteins with a variable number of common GDF-receptor (GFR) domains, which classically have 10 conserved cysteines, conserved cysteine–cysteine pairing, and 5 α-helices that form a rigid core between the first and last cysteines (204). In contrast to the homologous GDNF receptors and GAS1, GFRAL is fixed in the membrane through a transmembrane helix and not a GPI anchor. In existing models, GFR-domain 2 mediates interaction with the cognate GDNF and each receptor has a conserved RRR motif here that is important for ligand binding (22, 204). The receptors form GFRA homodimers upon their specific ligand binding (GDNF or GDF15) and recruit the receptor tyrosine kinase RET, which homodimerizes, autophosphorylates, and activates signal transduction. Although each GFRA has a known ligand, promiscuity between GFRAs and the GDNFs has been reported (205, 206), though GDF15 has not been observed to bind to any of the related GFRA receptors to date.

**Figure 3.**
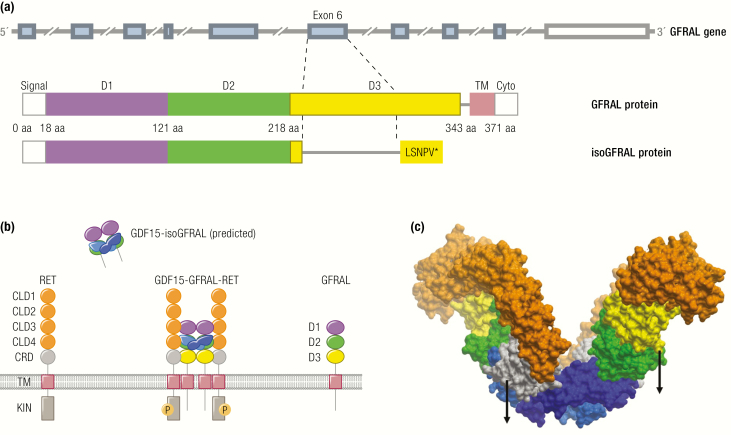
The GDF15-GFRAL-RET complex. The same color scheme is used to highlight the protein domains in all 3 panels. **A:** The gene structure of the human GRFAL alongside a schematic domain arrangement of the full-length GFRAL protein and its splice variant isoGFRAL. The coding exons are highlighted in light blue, with the 3’ UTR shown in white. GFRAL domains are labelled signal (signal peptide), D1-3 (GFR-like domain 1-3), TM (transmembrane domain), and cyto (cytoplasmic domain). The splice variant in which exon 6 is skipped as indicated and leading to a premature stop is predicted according to the homologous experimentally established mouse transcript. The resultant isoGFRAL lacks D3 and the downstream transmembrane domain and could function as a soluble GFRAL receptor isoform**. B:** Schema depicting GDF15, full-length GFRAL, the putative splice variant isoGFRAL, and RET. RET domains are labelled CLD1-4 (Cadherin-Like), CRD (cysteine rich), TM (trans membrane), and KIN (kinase). The activated (phosphorylated) receptor complex GDF15-GFRAL-RET is shown in the middle, while the putative isoGFRAL receptor is depicted extracellularly bound to GDF15. **C:** CryoEM structure of GDF15-GFRAL-RET ectodomain (PDB access code 6Q2J, surface visualization). The 2 GDF15 molecules in the dimer are distinguished in different shades of blue. Arrows indicate the linkers to the membrane for RET (left) and GFRAL (right) to the membrane (the remaining 2 are at the rear side).

GFRAL is encoded by the *GFRAL* gene, which in humans is found on the short arm of chromosome 6. It consists of 9 exons and encodes a 394 amino acid protein (Fig. 3A). A TATA-box motif is located 30 bps upstream of the transcriptional start site. Proximal to the TATA-box-predicted binding motifs for RUNX family transcription factor 1 (RUNX1), SRY-box transcription factor 5 (SOX5) and forkhead box Q1 (FOXQ1) have been described, while a proposed regulatory module consisting of 5 cis-elements is found approximately 700 bp upstream of the TSS (207). The full-length GFRAL protein has an N-terminal 18 amino acid signal peptide that targets GFRAL for membrane expression. At the C-terminus, amino acids 352–371 are predicted to form a transmembrane helix, while 372–394 form a cytoplasmic domain (Fig. 3A). The extracellular region of GFRAL consists of 3 cysteine-rich GFR-like domains (D1-D3). The cysteine pairing is conserved in D2 between GFRA1-4 and GFRAL, highlighting the importance of this structure in mediating the interaction between the GFRA-receptor family and their respective ligands. A hydrophobic pocket that is unique to GFRAL amongst the GFRAs mediates the interaction between GDF15 and GFRAL and confers specificity. Consistent with the existing models for GDNF–GFRA1 binding, D2 was necessary to mediate the GDF15–GFRAL interaction (22). What domains are required alongside D2 to mediate GDF15–GFRAL bonding is unclear from functional studies. One report using a flag-tagged GFRAL construct in an immunoprecipitation assay found that a mutant with D1 deleted could bind GDF15, whereas a second report using a flow-cytometry-based assay suggested that both D1 and D2, but not D3, were necessary for GDF15 to bind to GFRAL (36, 38). The exact detailed interactions are now clear from a cryogenic-electron microscopy (cryo-EM) study ((208); see below). In contrast to D2, the cysteine residues in D3 are poorly conserved between GFRAL and the canonical GFRAs, with a completely different schema for cysteine–cysteine pairing demonstrated in the crystal structure, the functional relevance of this is unclear (22). A splice variant of unknown functional importance has been described in the mouse transcriptome (207). The variant skips exon 6, resulting in a stop codon early in exon 7, which, if translated, would yield a GFRAL variant consisting of 2 GFR-like domains (D1–D2), lacking a transmembrane domain and last GFR-like domain (Fig 3A). It is, therefore, the membrane unbound and, according to the cryo-EM structure, ((208); see below) unable to activate RET even in the complex with GDF15. The expression level of the alternatively spliced GFRAL (isoGFRAL) was increased over 5-fold in the mouse embryo postnatally at day 0 and then markedly declined, becoming almost undetectable in the adult. Notably, the ratio of wild-type GFRAL to isoGFRAL increased to 3:1 at postnatal day 0 from 1:1 at embryonic day 17 (207). The homologous human alternative transcript can be predicted with high confidence but has not yet been described. The variant, if it is expressed in humans, will almost certainly retain GDF15 binding capacity but will be membrane unbound and unable to activate RET. It is conceivable that it acts as a decoy receptor blocking GDF15 activity in the embryo and protecting it against the circulating GDF15, leaving it to act solely on the mother’s brain (Fig. 3B). A similar optimised construct may find some medical potential as an alternative to therapeutic GDF15 antibodies.

### GFRAL signal transduction

Both receptor and coreceptor extracellular N-terminal parts are modular proteins; RET consists of 4 consecutive cadherin-like modules (CLD) and 1 cysteine-rich domain (CRD) and GFRAL of 3 GFR-like “D” domains (Fig. 3B). GDF15 binding to GFRAL is necessary for recruitment, binding, active arrangement of the dimer, and activation of the RET coreceptor (22, 36–38). Single amino acid substitutions in GDF15 that do not affect in vitro binding of GDF15 to GFRAL but do impair the antiobesity effect of the ligand suggest that GDF15 actively participates in the formation of the GFRAL-RET heterodimer via a direct interaction with RET (37). Recent publication of the cryo-EM structure of the extracellular portion of the GDF15-GFRAL-RET ternary complex provides a structural basis for the mechanistic understanding of this (208). Neither the ligand (GDF15) nor the coreceptor (GFRAL) alone is sufficient for activation of RET. The extracellular region of RET adopts a relatively rigid C-like shape stabilized by calcium ions bound at multiple sites. Its conformation changes very little when it clamps the coreceptor and the ligand. CLD3 domain of RET interacts with the GFRAL D3 domain and GDF15 is wedged between the CRD of RET and the second domain of the cadherin-like domain (CDL2) of RET (Fig 3C). The 2 halves of the ternary dimeric complex are brought together by the GDF15 dimer in the center. The receptor is activated upon the dimer formation. This model was substantiated by showing that single amino acid mutations in in either GFRAL and RET or GDF15 and RET interfaces prevented the complex formation (208).

Comparative analysis of the cryo-EM structures of GDF15-GFRAL-RET and the related GFRA-ternary complexes have provided unique insight into the activation of RET-dependent signaling (208). While the overall structure of all of the GFRA-ternary complexes is based on the same subunit architecture and domain interactions, the angle between the 2 wings of the ternary complex dimer varies between 57^o^ and 125^o^. The dimer formation brings 2 RET monomers in close association and triggers mutual phosphorylation of their intracellular domains (Fig. 3C). However, the NRTN-GFRA2-RET is capable of higher-order associations, as observed in cryoEM studies (208), but this is precluded in the GDF15-GFRAL-RET complex due to the sharper angle between the 2 wings. The higher-order ternary complex oligmers cannot be endocytosed, in contrast to the GFRAL-ternary complex that is endocytosed relatively quickly. The functional significance of these findings is 2-fold: (1) as the authors proposed, higher-order oligomerization may allow the cell to distinguish between GDF15-GFRAL dependent signaling and other RET ligands, and (2) if GFRAL and the related GFRA-receptors are coexpressed, RET could be sequestered at the surface in higher order oligomers and regulate GDF15 sensitivity. Conversely, GDF15-dependent endocytosis of RET could serve to inactivate signaling via the other GDNF-GFRA axis. It follows that if GFRAL and GFRA2 (for example) are coexpressed, genetic deletion of GFRAL may potentiate GFRA2-signaling via enhancing RET availability.

The activated GFRAL-RET heterodimer signals downstream to induce stimulatory phosphorylation of ERK (extracellular signal-related kinase), a finding that has been validated in vivo using GDF15 treatment of mice and coimmunostaining of GFRAL+ neurons with pERK (22, 37, 38). Akt and Phospholipase Cγ have also been shown to be activated by GDF15-GFRAL-RET signaling in vitro, but it should be noted that this data is exclusively derived from cells with ectopic expression of GFRAL +/- RET; it is unknown if endogenous GFRAL-RET signaling can activate these signaling modules in vivo (22, 37, 38). SMAD1/5/9, signal transducer And activator of transcription-3 (STAT3) or 5’ AMP-activated protein kinase (AMPK) were not phosphorylated in response to GDF15 treatment of HEK cells stably overexpressing human RET and GFRAL (38).

### GFRAL expression

As has been noted above, GFRAL is a hindbrain-restricted receptor expressed exclusively in the AP and NTS. An extensive survey of the whole rodent brain using RNA in situ hybridization (ISH) could only find evidence of GFRAL expression in the AP and NTS, where it was coexpressed with RET. These findings were confirmed by laser capture dissection and subsequent Nanostring mRNA analysis and immunohistochemistry (38). qPCR analysis of mouse hindbrain and subsequent localization studies by other groups corroborate the expression of *Gfral* in the AP and NTS and demonstrate that this expression distribution is conserved in rodents, monkeys, and humans (22, 36–38), though 1 group did not observe *Gfral* expression in the mouse NTS and an earlier report found evidence of more diffuse *Gfral* expression in the fetal mouse brain (36, 207). No evidence for the peripheral expression of *Gfral* in mice could be found despite 3 qPCR surveys by independent groups (22, 36, 37). In contrast, *GFRAL* mRNA was detectable in human adipose tissue and testes, but no GFRAL protein could be detected by immunohistochemistry (37). It should be noted that it is possible that GFRAL is rarely expressed in these tissues or in small subpopulations of cells that would be overlooked by bulk qPCR analysis. For example, exogenously administered GDF15 FC-fusion protein results in FC-immunoreactivity in the gut myenteric plexus, but this observation was made prior to the discovery of GFRAL, and the role of GFRAL in this phenomenon has not been tested (84). An additional consideration is that malignant transformation or other disease states could derepress GFRAL expression in peripheral tissues. These considerations notwithstanding, the existing evidence is in complete agreement that the expression of GFRAL, the GDF15 receptor, is limited to the brainstem.

### GFRAL mediates the effects of exogenous GDF15 on systemic metabolism

The dependence of GDF15 on GFRAL to exert its metabolic actions provide further evidence of the exclusivity of the GDF15–GFRAL interaction in vivo. GDF15 robustly cut food intake and induced weight loss in mice, rats, and monkeys, reducing weight in mice, for example, by around 10% after 10 days of treatment (22, 36–38). GDF15 treatment also improves glucose tolerance in both lean and obese mice (22, 36–38). None of these actions were observed in *Gfral*^-/-^ mice or in animals pretreated with a GFRAL-blocking antibody (22, 36–38). Two groups conducted pair feeding studies and concluded that the effects of GDF15 on weight loss were entirely explicable on the basis of its effect on food intake (37, 38). Others have suggested that GDF15 may also affect substrate utilization and ketogenesis, as GDF15 facilitates the development of ketosis upon 6 hours of fasting (22). However, with the experimental design used, an explanation based on reduced food intake contributing to ketogenesis cannot be excluded.

### Endogenous GDF15 and weight regulation: insights from GFRAL^-/-^ mice

When GDF15 is produced in excess, for example in cancer, it can have a profound effect on appetite and weight regulation (124). It is much less clear if endogenous GDF15 plays a role in physiological weight regulation in the lean state.

In studies by the Breit group, *Gdf15*^-/-^ mice were 6%–10% heavier than their wild-type counterparts and had increased lean and white adipose tissue mass (189). This difference in weight is observable as early as 5 weeks and, based on effects on weight in regression analysis of the whole cohort, increases on average by almost 1 gram over 1 year of life.

In 2 studies chow-fed *Gfral*^*-/-*^ animals had no difference in body weight or food intake, and energy expenditure, fat, and lean mass were unchanged (22, 38). Two other studies found that *Gfral*^*-/-*^mice had a paradoxical reduction in body weight compared to their age-matched controls. In 1 study, this phenotype was observed in both sexes of mice at 10 weeks of age but was more pronounced in males (36), while in a second study this reduction in body weight was only observed in males at 3–4 months and was inconsistent (37). The reason for the difference between *Gfral*^*-/-*^ and *Gdf15*^*-/-*^mice is unclear and there is no definitive explanation for the inconsistent phenotype observed in *Gfral*^*-/-*^ mice at present. Genetic background may partly explain the differences in *Gfral*^*-/ -*^and *Gdf15*^*-/-*^mice. The age-related weight gain observed in *Gdf15*^*-/-*^ mice on the chow diet was observed in a cohort of mice that had been extensively back-crossed to a C57BL/6 background, whereas *Gfral*^*-/-*^ mice were obtained from Taconic with a hybrid 129/SvEv–C57BL/6 background and were back-crossed to a C57BL/6N background twice in 1 of the studies (37). The inconsistent reduction in size and mass of *Gfral*^*-/-*^ animals may be related to the effects of GDF15 in utero; GFRAL may have a more extensive distribution in the mouse fetal CNS and, if this is true, then it may play additional developmental roles in mice (207). In studies of *Gdf15*^-/-^ mice, we should also consider the role of maternal genotype. If mothers are heterozygous for *Gdf15* then this could conceivably have effects on the development of the offspring.

In keeping with a protective role in the context of chronic caloric excess, it has been shown in 2 independent studies that *Gfral*^*-/-*^mice on a high-fat diet consume more food, gain more weight, and exhibit greater dysglycemia than their WT counterparts (22, 37), findings that are broadly consistent with findings from HFD-fed *Gdf15*^*-/-*^ mice (209). However, 2 other studies reported that *Gfral*^*-/-*^are of a similar weight to their WT counterparts when fed a high-fat diet (36, 38). It should be noted that circulating GDF15 was not measured in any of these studies, and it may be the case that circulating GDF15 may not be consistently elevated by all forms of high-fat feeding or that the impact of HFD-feeding is duration dependent

The Breit group hypothesized that compensatory changes occurring during the early development of *Gdf15*^-/-^ or *Gfral*^-/-^-null mice may lead to the underestimation of the impact of GDF15 signaling in adult mammals. To test this hypothesis, they used 2 independent approaches (210). Firstly, in mice with knock-in of the human *GDF15* gene replacing the endogenous mouse gene, a monoclonal antibody blocking human GDF15 was used. Antibody treatment of HFD-fed mice expressing human GDF15 resulted in increased food intake, fat mass and insulin resistance compared to control antibody or wild-type mice treated with the antibody to human GDF15. Secondly, a stereotactic injection of adenovirus (expressing a short hairpin RNA [ShRNA]) targeting *Gfral* into the AP and NTS was injected to achieve post-natal knockdown of *Gfral* in mice that were then subjected to HFD-feeding. On a HFD, *Gfral*-knockdown mice had increased bodyweight and adiposity compared to animals treated with a control ShRNA.

Considering the totality of the evidence, it seems unlikely that GDF15 is a physiological regulator of appetite in the normal state. It is not acutely regulated by over or underfeeding in humans and loss of GFRAL does not have any effect on food intake or adiposity in chow-fed mice (22, 36–38). Given that GDF15 is upregulated in obesity and that GDF15 can exert an anorectic action, it seems possible that GDF15 may be able to provide a regulatory brake on appetite and weight gain in states of chronic overnutrition. Whether this is the case or not likely depends on the magnitude of the increase in circulating GDF15 and the effect of the obese milieu on modulating sensitivity of GFRAL-RET signaling, its downstream effectors, and the neuronal circuitry downstream of GFRAL-neurons.

## GDF15 and the Activation of Aversive Central Pathways

Whether or not GDF15 provides a regulatory brake on weight gain in chronic overfeeding, it seems very unlikely that this is its primary evolutionary purpose. GFRAL expression is restricted to the hind brain in rodents, monkeys, and humans, and it is highly expressed in the AP, a sensory circumventricular organ with a highly permeable blood brain barrier, meaning it is exquisitely positioned to integrate endocrine signals from the peripheral blood and regulate behavior. Coupled with the fact that GDF15 is potently regulated by cellular stress, it seems likely that the primary role of GDF15 is to act as an endocrine message conveying somatic distress to the brain (47). What remains to be explored, however, is the full nature of the responses triggered by activation of GDF15-GFRAL-RET signaling and its adaptive purpose in the context of organismal stress.

Hsu and colleagues (22) found that treatment with GDF15 induces cFOS immunoreactivity in GFRAL-expressing neurons of the AP and NTS in a GFRAL-dependent matter within 1 hour. cFOS expression is also rapidly activated in the parabrachial nucleus, a site that receives afferent input from the NTS in humans and rodents (211, 212) and has been strongly implicated in the mediation of anorexia, malaise, and conditioned taste aversion (212–215). Consistent with engagement of this circuitry, 3 groups have now formally demonstrated that systemic (47, 216) and local (217) administration of GDF15 produces conditioned taste aversion (47, 216, 217).

Cytotoxic chemotherapy produces classically aversive responses. Consistent with GDF15 playing a role in this phenomenon, Hsu et al reported that cisplatin administration to wild-type mice resulted in elevated serum GDF15, suppressed food intake, and resulted in a 15% reduction in weight after 6 weeks. These effects were not seen in *Gfral*^*-/-*^animals (22). These findings have been confirmed by a second group working independently (217).

In addition to its effects on food intake and conditioned taste aversion exogenous, GDF15 increases kaolin consumption in rats, which is felt to be a physiological correlate of emesis (217, 218). Interestingly, GDF15 administration to obese rats markedly elevated kaolin consumption after 3 hours and preceded the onset of significant anorexia, which was not manifest until 24 hours after administration (217). In the same study, the authors administered GDF15 systemically to musk shrews, which have an emetic reflux. At the highest dose tested (1 mg/kg), GDF15 rapidly induced emesis, which preceded evidence of anorexia. The authors concluded that the anorexia observed with GDF15 administration was driven by nausea; however, it should be noted that a lower dose of 0.1 mg/kg did not illicit emesis in the musk shrew but resulted in comparable weight loss to the animals treated with the emetic dose.

To date, the principal focus of the central actions of GDF15 has been on measures relating to food intake and energy balance. In addition to effects on total caloric intake, GDF15 may influence food choice (84, 216). It seems likely that the activation of GFRAL-expressing neurons will have a much broader impact on the brain and its behavior. For example, there is some evidence that GDF15 results in changes in spontaneous physical activity (189, 209).

### GDF15, inflammation, and tolerance

Prior to the discovery of GRFAL, several lines of evidence from in vivo studies suggested an immunomodulatory effect of GDF15. Briefly, an examination of the role of GDF15 in myocardial infarction has suggested an anti-inflammatory action of GDF15. *Gdf15*^*-/-*^ mice exhibit increased mortality and infarct size in response to ischemia reperfusion injury. This is associated with an increased leucocyte number in histological sections of injured myocardium, and *Gdf15*^*-/-*^animals exhibit enhanced leucocyte adhesion and a rolling-in response to IL-1β treatment (142, 145). This study is corroborated by findings from an orthoptic model of glioma in mice, which used RNAi to reduce *GDF15* expression in a glioma cell line. Tumors formed from GDF15-depleted cell lines, which exhibited reduced macrophage and T-cell infiltration, although it was not determined if this was dependent on endothelial–leucocyte interaction (71). Further evidence that has been put forward to support an anti-inflammatory action of GDF15 is that transgenic mice that overexpress GDF15 exhibit reduced proinflammatory cytokines compared to their wild-type controls following treatment with lipopolysaccharide (219). In contrast, bone marrow chimeras with loss of GDF15 expression in the bone marrow compartment are protected from the development of atherosclerosis and exhibit reduced macrophage content in atherosclerotic plaques, suggesting a potential proinflammatory role for GDF15 in this setting (140).

How these studies could conform to a GFRAL-dependent model of GDF15 action is unclear. However, work by Luan et al has demonstrated a protective, central action of GDF15 in the setting of sepsis, which may be of relevance. Using a GDF15-blocking antibody to inhibit GDF15 activity, the authors demonstrated that GDF15 inhibition increased mortality in mouse models of bacterial and viral infection (184). The authors report that GDF15 maintains hepatic triglyceride output via selectively enhancing sympathetic outflow to the liver, thus defending a protective lower limit of triglycerides in the context of acute inflammation. In this study, relative hypotriglyceridemia induced by GDF15 blockade was associated with enhanced renal and cardiac toxicity, consistent with findings in GDF15 ^-/-^ mice after treatment with LPS (185). Importantly, Luan et al did not observe any changes in pathogen control or inflammatory cytokine production following GDF15 blockade, suggesting that the salutatory effects of GDF15 do not occur via enhanced immunity but rather via enhanced tissue tolerance. It is worth noting that the antibody used in this study was originally designed to antagonize human GDF15. While the authors validate their approach by demonstrating reduced cFOS immunoreactivity in the AP of anti-GDF15-treated mice, this was at a single timepoint and it is unclear if this approach blocked murine GDF15 for the entire duration of the study.

While these findings are intriguing, the opposite effect was observed in a polymicrobial model of sepsis undertaken in *Gdf15*^*-/-*^mice. In this study loss of *Gdf15* was associated with improved mortality and enhanced pathogen control. Moreover, enhanced recruitment of neutrophils to the site of inflammation was observed in *Gdf15*^*-/-*^mice; however, the exact mechanism was unclear (183).

The findings of Luan, if substantiated, raise a note of caution regarding the use of antagonists of GDF15-GFRAL in cachectic states, where patients are already prone to developing and succumbing to infection.

### Reconciling apparent peripheral actions of GDF15 with the restricted nature of GFRAL expression

A challenge to the coherence of the GFRAL-dependent mechanism of action of GDF15 is the vast body of pre-existing literature that suggests peripheral actions of GDF15. In particular, in vivo evidence that leveraged transgenic overexpression of *GDF15* or loss of endogenous *Gdf15* should be considered, as this would not be explained by contamination of recombinant GDF15.

One unifying mechanism could be that GDF15 exerts its observed peripheral regulatory actions via the hindbrain. The AP and NTS act to integrate inputs from endogenous and exogenous circulating factors and visceral afferents to regulate physiology and behavior such as autonomic tone, nausea and emesis, and satiety to food and fluids. The ability of GDF15 to regulate sympathetic outflow in the context of acute inflammation (184) is instructive, as it can be envisioned that in other contexts regulation of autonomic tone to adipose tissue and skeletal muscle could mediate proposed effects of GDF15 on energy expenditure (191).

A further unifying mechanism is that changes in behavior mediate some of the phenotypes observed in animals with gain or loss of GDF15-GFRAL-RET signaling. For example, altered food intake, reduced adiposity and accompanying changes in systemic metabolism could explain some of the protection from cancer seen in mice with overexpression of GDF15 and would be entirely coherent with GDF15 action via the hindbrain-restricted receptor GFRAL.

Further study of proposed peripheral actions of GDF15 are required to determine if they can be explained by a central action of GDF15 via GFRAL or if alternative mechanisms need to be explored.

#### Human genetics of GDF15.

SNPs in the vicinity of the *GDF15* gene fall into at least 7 independent haplotypes. A recent meta-analysis confirmed an association of all 7 SNP-labelled haplotypes with plasma concentrations of GDF15 (220), 3 of them with high genome-wide statistical significance in both discovery and validation cohorts (rs1054564, rs1227731, rs3195944). The haplotype block association was also very clear for 3 of them in a large study of plasma proteins in healthy individuals (221) and patients with cardiovascular disease (222).

These studies suggest that a SNP in each haplotype is causally associated with GDF15 plasma concentration and may in turn drive additional phenotypes. A potential mechanistic basis for the association between rs1054564 (red in Fig. 1a), which represents a G/C polymorphism in the GDF15 3’-UTR has been described. The minor allele, which is positively associated with GDF15, results in disruption of the 7merA1 pairing between miR-1233-3p, reducing the efficacy of GDF15 message targeting by this miRNA (16).

It should be noted that a common variant in the GDF15 coding region exists, which may confound interpretation of the genetic epidemiological studies cited above. rs1058587 (MAF = 24%) denotes a histidine to aspartic acid substitution at the sixth amino acid of mature GDF15 (202nd codon, H202D; Fig. 2). rs1058587 has been found to be associated with phenotypes such as the development of severe morning sickness in pregnancy (see below), prostate cancer, and venous thromboembolic disease in patients with rheumatoid arthritis (32,223,224). rs1058587 is in high LD (r^2^ > 0.9) with an annotated pQTL (rs45543339), determined using aptamer based technology, with the minor variant apparently associating with elevated plasma GDF15 (221). However, the association between rs45543339 and plasma GDF15 could not be confirmed with an independent validation assay, raising the suggestion that this finding may be spurious. Indeed, the polymorphic amino acid (H202D) encoded by rs1058587, which is in strong LD with the pQTL in question, is located near the mobile N-terminus of mature GDF15 and probably strongly contributes to its antigenicity. Moreover, the 2 variants can be distinguished through their distinct antigenicity (81), and it is possible that the current ELISA methods do not measure the concentration of the 2 mutants of GDF15 accurately. This outstanding methodological problem needs to be resolved urgently. In addition, the effects of rs1058587 on GDF15 bioactivity are unclear. No study has formally tested the potency of the H202D mutant with respect to GFRAL-dependent signaling or anorectic action. However, in prostate tumor xenografts, tumors formed from a cell line with the rs1058587 variant were smaller, secreted less GDF15, and exhibited less weight loss. However, these experiments are clearly confounded by differences in tumor size (198). The uncertainty regarding the functional and analytical significance of the rs1058587 variant makes it difficult to discern whether this SNP is causative for the associations described in human disease and confounds interpretation of the directionality of effect of GDF15 in genome-wide association studies (GWAS).

Recently, SNPs associated with circulating GDF15 have been used as instruments to undertake Mendelian randomization analysis and test the causal associations of GDF15 with various disease processes. Using a 5 SNP instrument variable (which explained <21% of variance in circulating GDF15), Au Yeung et al were unable to provide any evidence that GDF15 was related to the development of type 2 diabetes, HbA1c, blood pressure, or BMI (225). The genetic instrument was associated with coronary artery disease and breast and lung cancer, but these findings could not be replicated in the validation cohort. Similarly a 3 SNP instrument could not find any evidence to support a causal association to support a relationship between GDF15 and cardiometabolic disease (226).

The above studies suffer from some limitations but most notably the differences in GDF15 are modest compared to the alterations seen in human disease, and there is uncertainty regarding the validity of GDF15 measurements by current methods in the presence of the common rs1058587 variant.

An alternative approach to uncovering the functional importance of human GDF15 in human health and disease is to identify coding variants that may alter GDF15 structure and examine their association with human phenotypes. rs1058587 (H202D) has been discussed above. Three further common (MAF > 1%) polymorphisms exist: V9L rs1059519 27%, S48T rs1059369 27%, and S164SS (duplication, in frame insertion) rs199673307 2.2%. Our molecular modeling does not indicate that the amino acid replacements introduce significant functional changes in the three-dimensional structure. V9L is a very conservative replacement in the segment preceding the transmembrane helix in the signal peptide, and S48 lies in a mobile loop on the surface. Moreover, none of them are conserved in evolution (Fig. 2A, Supplementary Fig.1A available at (31)).

## GDF15 and Pregnancy

The first indication that GDF15 may play a specific role in pregnancy came from the findings of high levels of expression in the placentas of PTGF-β (2) and PLAB (5), both earlier synonyms for GDF15. Subsequent research (34, 227, 228) has confirmed that GDF15 is highly expressed in the trophoblast, that circulating GDF15 is markedly elevated in human pregnancy, and is present in high concentrations in amniotic fluid. GDF15 has been suggested to be a biomarker of miscarriage and of pre-eclampsia, leading to speculation that it might exert local actions in the placenta (229, 230), something that would be inconsistent with the exclusively central expression of its sole known receptor. Rather, emerging evidence suggests that GDF15 plays an important role in nausea and vomiting of pregnancy and hyperemesis gravidarum (HG).

### GDF15 and nausea and vomiting of pregnancy

GDF15 is expressed at high levels in human syncytiotrophoblast and maternal GDF15 levels rise markedly in the first trimester of pregnancy and continue to rise, albeit more slowly, through the rest of pregnancy (231). This fact coupled with the known anorectic action of GDF15 via GFRAL in the medullary chemoreceptor trigger zone have led to the suggestion that GDF15 may be involved in nausea and vomiting in pregnancy (NVP) and HG.

Nausea and vomiting in pregnancy, colloquially (but misleadingly (232)) referred to as morning sickness, affects approximately 70% of pregnancies (233). Nausea and vomiting in pregnancy is a feature of most healthy pregnancies and in its mildest form it does not have implications for the development or long-term health of the fetus. At its most severe it is known as HG (hyperemesis gravidarum) and is associated with intractable vomiting, dehydration, weight loss, and electrolyte derangement. The consequences of HG range from psychological morbidity and lost income (234, 235) to impaired nutrition, termination of wanted pregnancies, and adverse obstetric outcomes (236–238).

Using a large prospective cohort study of pregnancy, the Cambridge Baby Growth Study, our group have provided evidence to support the assertion that GDF15 is implicated in NVP and HG (231). Circulating GDF15 levels (at approximately 15 weeks gestation) were significantly elevated in women who experienced vomiting in the second trimester or who used antiemetics in pregnancy.

It should be noted that GDF15 is elevated >10-fold in normal pregnancy compared to the nongravid state, and our own work has demonstrated that GDF15 is present at an average concentration of ~10 600 pg/ml in women who do not experience any NVP. To our knowledge there is no other scenario in human health where GDF15 circulates in such high concentrations, and the amount of circulating GDF15 in pregnancy is commensurate with those seen in anorexia/cachexia syndromes. The modest elevation in GDF15 seen in women with nausea and vomiting could of course be a secondary phenomenon. In this regard important genetic evidence has directly implicated GDF15 in the pathogenesis of HG. Fejzo et al used customers of 23andMe to examine the genetic risk factors for NVP and HG (223). A genome-wide association scan for loci associated with HG identified an association signal at Chr19p13.11, where GDF15 is situated. The lead SNP rs45543339 is situated downstream of *GDF15* and is associated with a 33% reduction in relative risk of HG. Moreover, this SNP was in high linkage disequilibrium with a common missense variant in GDF15, rs1058587, which was associated with a similar relative risk reduction to the lead variant. The same locus was identified in an independent genome-wide scan and was confirmed in an independent replication cohort where cases were defined as women who required IV fluids for HG and controls who reported “normal NVP” (223). A second replication cohort, in which cases were defined as women with HG requiring total parenteral nutrition and controls who did not experience NVP in at least 2 pregnancies, confirmed the association, but this was not found to be significant after adjustment for multiple testing.

In addition, the SNPs associated with HG in this study represented the maternal genome and do not represent the genotype of the placenta. This raises at least 2 scenarios which are not mutually exclusive: (1) placental GDF15 is the key determinant of plasma GDF15 in pregnancy and the effect size observed in this study is diluted by pregnancies in which the fetus does not inherit the risk allele from the mother, and (2) extra-placental GDF15 makes a meaningful contribution to plasma GDF15 in pregnancy and the risk alleles modulate GDF15 risk even when the genotype of the mother and her pregnancy are discordant with respect to the causative SNPs. Further work delineating the risk of HG by maternal and fetal genotype and the effects of these SNPs on circulating GDF15 in pregnancy will be necessary to resolve these issues. It should be reiterated that the rs1058587 variant which is protective for HG (223) is associated with elevated GDF15 levels in a GWAS of the human plasma proteome but there were discrepancies in GDF15 levels in the 2 assays used in the study, and the effect of this variant on GDF15 antigenicity and bioactivity is unclear (221).

If, as we believe, GDF15 acts solely through GFRAL in the hindbrain, why are circulating levels so high in pregnant women from the very early stages of pregnancy? The robust association of SNPs close to GDF15 with severe NVP, a phenomenon which is present to a milder degree in the majority of pregnant women, and the almost universal reports of changes in food preferences in pregnancy provides support for the idea that high levels of maternal GDF15 may influence maternal appetite, food preference, and food intake. Why would this have been selected for during our evolution? One hypothesis might be as follows. The human fetus is highly susceptible to teratogens, especially in the first trimester. During most of human evolution, as hunter–gatherers, daily life involved the ingestion of calories from a wide variety of sources, many of which contained chemicals potentially damaging to fetal development. Might high GDF15 levels sensitize the CNS and encourage avoidance of certain foodstuffs? For example, pregnant women frequently report an intolerance of tea and coffee, both of which contain multiple alkaloids (239, 240). Severe NVP and HG may represent the extreme end of a normal spectrum of appetite change and despite their obvious adverse effects on the mother may reflect that lesser degrees of appetite change have been actively selected for during evolution.

Aside from NVP and HG, altered food perception and eating behavior is relatively common in pregnancy. Pica, voluntary consumption of non-nutritive substances, occurs in almost 30% of pregnancies, according to one meta-analysis (241). Why and how Pica occurs in pregnancy is unclear, and it initially seems incongruent with a susceptibility to food aversion mediated by GDF15. However, it has been proposed that it may protect the fetus from ingested toxins (242). Geophagy—eating soil or earth—is a common form of pica and has been proposed to reduce the absorption of harmful toxins. Treatment of rats with cisplatin results in the suppression of food intake but increases the consumption of the kaolin clay, reduces associated morbidity, and is a physiological correlate of nausea in rodents, which do not have an emetic reflex (218). Indeed, GDF15 induces kaolin consumption in rats (217). It is unclear if GDF15 mediates pica in humans, but if it does, the paradoxical increase in appetite for an exogenous agent that may have a detoxifying purpose would provide additional evidence for GDF15 as a mediator of toxin avoidance in pregnancy.

It is also possible that GDF15 has other central effects through, for example, the autonomic or neuroendocrine axes, that might facilitate maternal adaptation to the pregnant state, but this has not been explored.

## Therapeutic Implications

### GFRAL antagonism

Antagonism of the GDF15 hormonal axis has been achieved in preclinical settings with blocking antibodies to both GDF15 and GFRAL. The potential therapeutic utility of GDF15-GRFAL antagonism has been demonstrated most clearly in the setting of cancer cachexia, where a blocking antibody to GDF15 prevented the cancer cachexia associated with xenograft tumors in mice (124). The association of raised circulating levels of GDF15 with cancer cachexia make this an obvious area for therapeutic investigation. Modulation of the GDF15-GFRAL axis may also be beneficial for the treatment of other anorexia/cachexia syndromes, for example, end-stage renal disease, heart failure, and COPD, which are all associated with anorexia/cachexia syndromes that confer an adverse prognosis. In addition, advanced age and frailty are associated with anorexia and sarcopenia and are associated with elevated circulating GDF15. It is tempting to speculate that in this setting GDF15-GFRAL antagonism could lengthen the “health span” in adults (112, 113).

Mice lacking GFRAL are relatively resistant to the suppressive effects of cisplatin on food intake (22), with obvious implications for the prevention of chemotherapy-associated nausea and vomiting. Drug research efforts in this arena have led to the development of therapies, including a range of 5-hydroxytryptamine-3 (5HT3)-antagonists and the NK1 antagonist aprepitant. Despite the use of these agents, treatment failure, defined as emesis or the need for rescue medication, is common (243). GFRAL antagonism presents an exciting opportunity to add to the current pharmacological approaches to improve the experience of patients undergoing cytotoxic chemotherapy. While data regarding the effects of GFRAL antagonism in humans has not yet been published, a phase 1 study of a GFRAL-blocking antibody (NGM120) in healthy participants has been completed (NCT03392116) and a further phase 1 trial in advanced solid malignancy is ongoing (NCT04068896).

As discussed above, both genetic and biochemical evidence support the notion that GDF15 is causally related to severe NVP and HG. Could a therapy based on antagonism of the GDF15–GFRAL axis be both effective and safe in the treatment of these conditions? The considerable overlap in GDF15 concentrations between asymptomatic women and those with severe NVP suggest that factors other than the high GDF15 levels themselves must be involved. Indeed, the seminal genetic study in the area did highlight several other genes (223). However, it is plausible that while hyperactivation of GFRAL-RET may not be sufficient for the production of HG, it might be necessary. There are numerous daunting challenges to be overcome before this hypothesis could be tested as a prelude to the ultimate development of a GDF15-GFRAL blocking drug for the treatment of hyperemesis. There are no preclinical species that develop HG. Safety issues concern both the mother and the developing fetus with limited precedents for drug licensing in the first and second trimester. However, if it were shown to be effective, there are reasons why GFRAL antagonism might prove safer than current treatments that are resorted to in severe cases of HG. Firstly, an antibody to GDF15 or GFRAL could be readily modified to minimize transplacental transport and exposure to the fetus—much like has been done with anti-TNF-α therapies for use in pregnancy (244). Secondly, the expression of *GFRAL* in a normal human fetus appears extremely low, bordering on undetectable (Prof. Neil Hanlem, University of Manchester, personal communication), so the risk of mechanism-based toxicity to the fetus, even if some antibody did cross the placenta, would be very low.

### GFRAL agonism

The therapeutic potential of GFRAL agonism has been demonstrated in preclinical studies in mice, rats, and monkeys and modified; long acting molecules have been developed with impressive preclinical effects (22, 36–38, 84). GDF15 also seems to have additional effects on food preference (84, 216). In addition, GDF15 treatment has been demonstrated to exert synergism when combined with the established antiobesity agents, GLP-1R analogues (216).

Based on published observational data demonstrating that GDF15 was a biomarker of metformin use (245), our group tested the hypothesis that metformin may actually mediate its weight-reducing actions via GDF15. The weight-reducing effects of metformin are increasingly recognized to make an important and sustained contribution to metformin’s action, particularly in the prevention of Type 2 diabetes (246, 247). In a post hoc analysis of a randomized controlled trial of metformin in patients with obesity and proven coronary artery disease (248), metformin administration led to substantial and sustained elevations in circulating levels of GDF15. Plasma samples were collected and analyzed at baseline and after 6, 12, and 18 months of treatment. In metformin-treated subjects, GDF15 concentrations were in the region of 1800 pg/ml after 6, 12, and 18 months of treatment, representing an increase of GDF15 of around 50%, relative to baseline (60). This corresponded to an increase in plasma GDF15 of almost 40% relative to placebo-treated controls. In the same paper, our group reported a post hoc analysis of a small, short-term, double-blind, crossover, placebo-controlled trial of metformin where compliance was in excess of 90% throughout the study (249). Circulating GDF15 was 2.5-fold higher after 2 weeks of metformin treatment compared to circulating levels measured after 2 weeks of placebo treatment. Mouse studies demonstrated that the ability of metformin to prevent weight gain in mice subject to a high fat diet was completely blocked in mice lacking either GDF15 or GFRAL. Moreover, mice rendered obese by high-fat feeding lost weight when given metformin, but this was blocked by a GFRAL blocking antibody. Whilst all of the effects of Metformin on weight and body composition seemed to be GDF15-dependent, metformin retained its glucose- and insulin-lowering effects in the absence of GDF15. The source of GDF15 induced by metformin was mainly the small intestine, colon, and kidney, as metformin treatment induced *Gdf15* mRNA at these sites but not in adipose tissue, liver, or skeletal muscle. Importantly, these findings have been independently replicated and published almost simultaneously by the Steinberg group. Using *Gdf15*^*-/-*^ mice they showed that endogenous GDF15 was required for the weight-lowering actions of metformin in diet-induced obesity; however, they did not explore the source of GDF15 in vivo (76).

It should be noted that in contrast to our work, the authors of this study found that the glucoregulatory actions of chronic metformin treatment in diet-induced obesity were dependent on the presence of GDF15. The reason for this discrepancy is not clear but the lack of an effect on glucose metabolism in our study may relate to mode of metformin administration, route of glucose tolerance testing (oral gavage in our study vs. intraperitoneal), or the specific method used to ablate GDF15-GFRAL-RET signaling. Interestingly, the elevation of circulating GDF15 observed with metformin treatment in both studies was much more modest in chow-fed than HFD-fed mice, suggesting that obesity somehow potentiates GDF15 induction in response to metformin. This study also demonstrated a small effect of metformin on food intake in chow-fed mice in both *Gdf15*^*+/+*^ and *Gdf15*^*-/-*^ mice, suggesting that the anorectic actions of metformin in lean and obese animals occur via different mechanisms.

In addition to the obvious mechanistic interest of these studies, they are of immediate translational relevance, as the fact that metformin chronically elevates GDF15 is reassuring for the development of GDF15-GFRAL agonists as antiobesity agents given the safety profile of metformin over 60 years of widespread use in man.

### GDF15: potion or poison?

There is an unhelpful tendency in medical science to eulogize or demonize biological pathways. Such an oversimplistic view seems particularly inappropriate for GDF15, where context is all important.

On the one hand, it is highly likely that elevated levels of GDF15 actively participate in driving nausea, emesis, and cachexia syndromes in the context of cytotoxic chemotherapy, cancer, and other severe systemic diseases.

However, it is unlikely that GDF15 evolved to make us ill. It surely must have served some adaptive functions during our evolutionary history. Its response to ingested toxins and its perception as aversive may well have alerted us to potentially harmful chemicals in plants or animals encountered while we were hunter–gatherers. Emerging evidence of actions in the context of acute inflammation suggest that it may have adaptive functions that remain relevant today.

To complicate matters further, our increasingly obesogenic environment may yet restyle this aversive, potentially emetogenic substance as a therapy for overnutrition-related diseases

Many puzzles remain to be solved. Are the elevated GDF15 concentrations found in conditions such as ageing and frailty, with chronic renal and cardiovascular diseases playing an active role in the pathogenesis of these disorders? If not, are they part of a helpful adaptive response to disease or are they simply a biomarker?

Disentangling GDF15 as a driver of disease to be inhibited or as a protective reactive change to be potentiated will continue to be difficult, but detailed mechanistic understanding afforded by the discovery of GFRAL will be invaluable while the therapeutic potential of targeting the GDF15-GFRAL-RET axis for the treatment of human disease is explored.

## Summary and Future Directions

GDF15 is a hormone that signals states of somatic distress to the brain via the receptor GFRAL-RET, which is highly anatomically restricted. It has clear anorectic actions and emerging data suggests that it may have aversive properties. We have proposed that a major adaptive consequence of stress-induced regulation of GDF15 is a learned aversion to toxic environmental stimuli and have extended this to the setting of pregnancy whereby placental GDF15 may facilitate hypervigilance against harmful environmental agents. The discovery of GFRAL as the GDF15 receptor has invigorated interest in this axis as a potential therapeutic target. The most obvious route to clinical utility is in the use of GDF15 antagonists in chemotherapy-induced nausea and vomiting and in various forms of cachexia associated with high circulating GDF15. Some support for the notion that chronic elevation of GDF15 action might be safe and effective in obesity is provided by studies demonstrating the chronic elevation of GDF15 by metformin and the importance of this action for metformin’s beneficial effects on weight.

Our understanding of the physiological function of the GDF15-GFRAL-RET axis and its role in human disease remains in its infancy, and a number of key questions remain unanswered. While existing evidence has clearly defined a role for GDF15 as an anorectic peptide, it is likely that further physiological actions of GFRAL-RET signaling exist and remain to be defined. Identifying these actions and determining if they can account for some of the phenotypes previously attributed to a peripheral action of GDF15 will provide further mechanistic insight into the role of the GDF15-GFRAL-RET axis in human disease. Exploring the genetic epidemiology of circulating GDF15 may be a useful strategy to further understand the effects of GDF15 in humans, but the utility of this approach is likely to be limited until we can fully understand how the common H202D variant affects circulating GDF15 independent of antigenicity. The fact that metformin exerts its regulatory effect on weight via GDF15-GFRAL-RET provides proof of principle for GFRAL agonism as a therapeutic strategy in obesity. However, the efficacy and tolerability of more potent agonism aiming to achieve weight loss comparable to existing obesity therapies remains to be defined.

## Additional Information

***Disclosure Summary:*** S.O.R. is an employee of the University of Cambridge and has provided remunerated consultancy services to the following pharmaceutical and biotechnology companies: Pfizer, AstraZeneca, and Novo-Nordisk GSK, and ERX. S.M.L. and V.S. have no relevant conflicts of interest to declare.

***Data Availability:*** All data generated or analyzed during this study are included in this published article or in the data repositories listed in References.
